# S-phase checkpoint protects from aberrant replication fork processing and degradation

**DOI:** 10.1093/nar/gkaf707

**Published:** 2025-07-30

**Authors:** Iván Núñez-Martín, Lucy S Drury, María I Martínez-Jiménez, Luis Blanco, John F X Diffley, Andrés Aguilera, Belén Gómez-González

**Affiliations:** Andalusian Center of Molecular Biology and Regenerative Medicine, Universidad de Sevilla-CSIC-Universidad Pablo de Olavide, Seville 41092, Spain; Departamento de Genética, Facultad de Biología, Universidad de Sevilla, Seville 41012, Spain; Chromosome Replication Laboratory. The Francis Crick Institute, 1 Midland Road, London NW1 1AT, United Kingdom; Centro de Biología Molecular Severo Ochoa, CSIC-UAM, Madrid 28049, Spain; Centro de Biología Molecular Severo Ochoa, CSIC-UAM, Madrid 28049, Spain; Chromosome Replication Laboratory. The Francis Crick Institute, 1 Midland Road, London NW1 1AT, United Kingdom; Andalusian Center of Molecular Biology and Regenerative Medicine, Universidad de Sevilla-CSIC-Universidad Pablo de Olavide, Seville 41092, Spain; Departamento de Genética, Facultad de Biología, Universidad de Sevilla, Seville 41012, Spain; Andalusian Center of Molecular Biology and Regenerative Medicine, Universidad de Sevilla-CSIC-Universidad Pablo de Olavide, Seville 41092, Spain; Departamento de Genética, Facultad de Biología, Universidad de Sevilla, Seville 41012, Spain

## Abstract

Replication stress, a hallmark of cancer cells, is detected by checkpoint mechanisms that trigger a range of cellular responses. Among these, the preservation of replication fork integrity is crucial for ensuring survival in the presence of DNA damage. In budding yeast checkpoint mutants, DNA damage leads to irreversible replication fork arrest and subsequent cell death, though the underlying mechanism remains unclear. Our study reveals that several DNA processing factors, including Rad51, the Rad5 HIRAN and helicase domains, and the catalytic activity of Mus81, contribute to this lethality. Nevertheless, their roles are masked by their essential functions in cell survival after damage removal. Additionally, we show that these factors, along with Exo1, drive the gradual degradation of nascent DNA at replication forks upon DNA damage. Notably, this degradation can be mitigated by expression of human PrimPol, which is absent in yeast. These findings suggest that the essential role of S-phase checkpoints upon DNA damage is to safeguard stalled replication forks from aberrant processing, thereby preserving nascent DNA integrity.

## Graphical Abstract

**Graphical Abstract:**
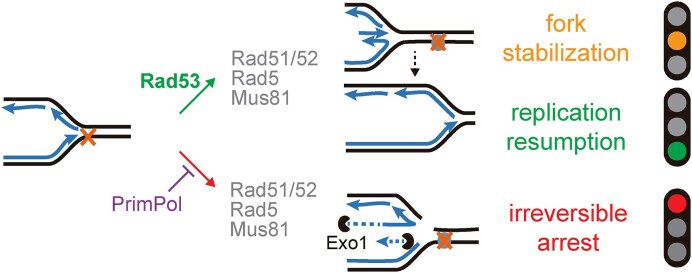

## Introduction

Precise duplication and transmission of genetic material to daughter cells are essential for cell survival, with replication stress widely recognized as a hallmark of cancer cells due to its detrimental impact on genome integrity [1]. Replication stress-inducing agents impair DNA synthesis by replicative polymerases without affecting DNA unwinding by the replicative helicase, thus uncoupling the two activities and causing an accumulation of single-stranded DNA (ssDNA). This ssDNA leads to the activation of the S-phase checkpoint, the main players of which in the budding yeast *Saccharomyces cerevisiae* are the apical transduction and effector kinases Mec1 and Rad53 (human ATR and CHK1 homologues). Checkpoint activation prevents further uncoupling between leading and lagging strand synthesis and triggers several responses to ensure cell survival [2–5]. These responses not only block cell cycle progression but also regulate origin firing, fork progression, gene expression, and DNA repair.

Among checkpoint functions, the upregulation of dNTP pools via degradation of the ribonucleotide reductase inhibitor Sml1 is essential for survival [6–10]. However, even when this function is overcome by genetic alterations such as the deletion of *SML1*, yeast checkpoint mutants become extremely sensitive to DNA damaging agents that cause replication stress, such as hydroxyurea (HU) or the alkylating agent methyl methane-sulfonate (MMS) [11, 12]. In addition, they accumulate DNA damage repair foci, which can be observed by the fluorescent tagging of the recombination protein Rad52 [13]. In checkpoint-deficient cells, a significant amount of replication forks irreversibly arrest, as originally observed by density-transfer experiments [14]. The regulation of gene expression and the cell cycle block were ruled out to be the cause of such a fork arrest since neither inhibiting protein production by cycloheximide nor restraining mitotic entry with nocodazole were able to suppress the lethality of HU in *rad53Δ* cells [14]. Also, preventing the inhibition of late origin firing was not enough to suppress the lethality [15]. 2D-gel experiments and ssDNA analyses by electron microscopy suggested that DNA can undergo degradation at forks [16–19]. This was also supported by the detection of differential polymerase usage at the DNA leading strand [20]. However, there is no direct evidence for nascent DNA degradation in yeast.

In order to uncover factors involved in irreversible replication fork arrest, efforts have been made to identify suppressors of the *rad53Δ* sensitivity to DNA damage [21–23]. Among them, deletion or phosphomimic mutations in the Rad53 target Exo1 slightly suppress the MMS sensitivity of checkpoint mutants [21, 24]. Mutations that suppress the DNA damage sensitivity of *rad53Δ* cells have been found in genes of other Rad53 target proteins such as RNA metabolism factors and nucleoporins, but this suppression was only observed at low doses of HU [22]. Stronger suppression was described in mutants of the Rpd3L histone deacetylase complex even at high doses of MMS although this effect could be indirect [23]. The recent observation that mutation of the Rad53 phosphorylation sites in Mrc1 that regulate fork rate can slightly rescue viability of checkpoint mutants at very low doses of HU or MMS argues that restraining fork speed might contribute to fork stability, but this may not be the only or main suppressor pathway [25].

In this paper, we found that Rad51, Rad5 HIRAN and helicase domains, and Mus81 catalytic activity contribute to the irreversible arrest caused by MMS in *rad53Δ* cells but importantly, these proteins also have a role in cell survival once the damage is removed. In addition, *MRE11* or *RAD50* overexpression can partially counteract fork arrest independently of its nuclease activity. The effect of MMS treatment on the loss of previously incorporated BrdU allowed us to directly measure nascent DNA degradation in checkpoint-deficient cells and reveal that this is prevented by the absence of Rad51, Rad5, Mus81, or Exo1 but not by the loss of Mre11. The process of irreversible replication fork arrest is thus likely driven by a template-switching reaction, such as fork reversal, and nucleolytic cleavage, mediated by Mus81 but not by Mre11. In addition, we found that expression of human PrimPol, which can rescue stalled replication forks by repriming the leading strand [26, 27] (reviewed in [28]), prevented nascent DNA degradation and enhanced survival of yeast checkpoint mutants upon exogenous DNA damage, thus suggesting that PrimPol can function in yeast. Altogether, our study uncovers key factors involved in irreversible fork arrest and supports a model in which checkpoint deficiency leads to aberrant processing of forks stalled in response to endogenous or exogenous agents.

## Materials and methods

### Yeast strains and media

Yeast strains used in this study are listed and described in Supplementary Table S1.

Media used in this study: YPAD (1% yeast extract, 2% peptone, 2% glucose, 20 mg/l adenine), YPAGal (1% yeast extract, 2% peptone, 2% galactose, 20 mg/l adenine), YPARaf (1% yeast extract, 2% peptone, 2% raffinose, 20 mg/l adenine), SC (0.17% yeast nitrogen base without amino acids nor ammonium sulfate, 0.5% ammonium sulfate and supplemented with amino acids, 2% glucose), and SPO (1% potassium acid, 0.1% yeast extract, 0.005% glucose). Solid media were prepared adding 2% agar before autoclaving. For MMS-containing plates, the drug was added to the melted agar just before pouring the plates that were dried with the lid in place for 24 h before use. Plates were sealed with parafilm during incubation.

Yeast strains were freshly defrosted from glycerol stocks and grown at 30°C using standard practices. All experiments were performed at 30°C unless specified.

### Plasmids

Plasmids pRS306-GAL1,10–3xFlag-MUS81 carrying the *MUS81* gene fused to the *GAL1,10* promoter [29], pML107 for CRISPR genome editing [30], pWJ1344 carrying Rad52-YFP fusion [13], pRS313, pFA6a-*hph*NT1, pFA6a-*kan*MX4, pFA6a-*nat*NT2, and pYM-*hph*NT1 used for gene replacement [31, 32], pYM-N26 and pYM-N27 [32] used for *GALL* promoter replacement have been previously described.

pBG012 carrying *RAD5* gene was generated by cloning *PvuII-HindII*-digested *RAD5* ORF into pFA6A-*hph*NT1. pBG013 carrying the mutated version *rad5-QD* was generated by directed mutagenesis of plasmid pBG012.

pRS306-GAL1,10-RAD5 and pRS306-GAL1,10-PRIMPOL for *RAD5* and PrimPol expression were generated by replacing the *3xFlag-MUS81* gene of plasmid pRS306-GAL1,10–3xFlag-MUS81 with the *RAD5* and *PRIMPOL* coding sequences, respectively, following In-Fusion HD EcoDry cloning kit (Takara Bio) procedure. *RAD5* ORF was amplified by polymerase chain reaction from plasmid pBG012 using primers RAD5.InFus.fw and RAD5.InFus.rv and *PRIMPOL* coding sequence was amplified from plasmid pET16-His-PRIMPOL [33] using primers PRIMPOL.InFus.fw and PRIMPOL.InFus.rv. In both cases, plasmid pRS306-GAL1,10–3xFlag-MUS81 was linearized without *3xFlag-MUS81* sequence using primers pRS306-G-MUS81.InFus.fw and pRS306-G-MUS81.InFus.rv. Plasmids pRS306-GAL1,10-PRIMPOL-AxA and pRS306-GAL1,10-PRIMPOL-CH were generated using the same strategy than pRS306-GAL1,10-PRIMPOL.

pRS304-GAL1,10-POL1,POL12 and pRS306-GAL1,10-PRI1,PRI2 for expression of all the subunits of the Polα/Primase complex were generated by cloning coding sequences of POL1 and POL12 into AscI-KpnI and SgraI-NotI of pRS304-GAL1,10 and PRI1 and PRI2 into AscI-XhoI and SgraI-NotI of pRS306-GAL1,10, respectively.

pRS303-GAL1,10-PRIMPOL was generated by replacing *URA3* marker of plasmid pRS306-GAL1,10-PRIMPOL by *HIS3* gene, following In-Fusion HD EcoDry cloning kit (Takara Bio) procedure. *HIS3* gene was amplified from plasmid pRS313 using primers HIS3.pRS306.FW.InFus and HIS3.pRS306.RV.InFus, and plasmid pRS306-GAL1,10-PRIMPOL was linearized without *URA3* marker using primers pRS306.URA3.FW.InFusion and pRS306.URA3.RV.InFusion.

Plasmids pML107-mre11-H125N, pML107-mus81-dd, pML107-Rad5-I916A, pML107-Rad5-HIRAN used for generation of point mutants with CRISPR technology were obtained by cloning single guide RNAs (sgRNAs) into *BclI-SwaI*-digested pML107 [30]. sgRNAs were obtained by hybridising primers Mre11-H125N-CRISPR1 and Mre11-H125N-CRISPR2, Mus81-dd-CRISPR3 and Mus81-dd-CRISPR4, gRNA.Rad5-I916A.1 and gRNA.Rad5-I916A.2, and Rad5Hiran1 and Rad5Hiran2, respectively.

All primers are listed in Supplementary Table S2.

### Analysis of Rad52-YFP foci

To detect Rad52 foci, cells were transformed with plasmid pWJ1344 expressing RAD52-YFP, fixed with formaldehyde and visualized with a Zeiss AX10 fluorescence or a Leica DM6000 microscope with a 100×/1.4 oil objective. Foci in S/G2 cells were counted in asynchronous or in α-factor synchronized cultures that were released for 75 min. For MMS treatment, exponentially growing or G1-synchronized cultures were treated with MMS at the concentration indicated and incubated for 60 or 90 min before counting. For foci recovery experiments, cells were arrested in G1 with α-factor, released in S phase in the presence of MMS and nocodazole (5 μg/ml) for 1 h and subsequently treated with 2.5% sodium thiosulphate to inactivate the MMS. Cells were then washed and released into fresh nocodazole-containing medium without MMS for up to 4 h to allow time for foci repair without entering the next cell cycle. A total number of 100 cells were analysed in each experiment for each time point. Number of cells with 1, 2, or >2 foci are represented but statistical analyses were performed with the total number of foci. The mean values and standard deviation of the total number of foci resulting from three or four experiments performed with independent transformants are shown.

### Western blot

Western blots were performed using standard procedures with the following primary antibody dilutions: 1:1000 rabbit polyclonal anti-Rad53 antibody (JDI4), 1:2000 rabbit polyclonal anti-Rad51 antibody (ab63798, Abcam), and 1:5000 anti-histone H2A polyclonal antibody (39 235, Active motif) for Supplementary Fig. S1; 1:2000 rabbit polyclonal anti-Rad53 antibody (ab104232, Abcam), 1:2000 rabbit polyclonal anti-beta actin antibody (ab8227, Abcam), and 1:5000 rabbit polyclonal anti-PrimPol antibody [26] for Fig. 7A and B; 1:10 000 mouse monoclonal anti-Pgk1 antibody (22C5D8, Invitrogen), 1:5000 rabbit polyclonal anti-histone H4 antibody (ab7311, Abcam), 1:5000 rabbit polyclonal anti-PrimPol antibody [26], and 1:2000 rabbit polyclonal anti-Rfa1/Rpa1 antibody (ab221198, Abcam) for chromatin fractionation experiments in Fig. 7C and Supplementary Fig. S6A and B. The secondary antibody dilutions were: 1:5000 secondary goat anti-rabbit IgG-horseradish peroxidase (HRP) antibody (A6154, Sigma–Aldrich) and 1:5000 secondary goat anti-mouse IgG-HRP antibody (A4416, Sigma–Aldrich). All signals were acquired and quantified in a Chemidoc MP imaging system and Image Lab software v6.1.0 (Bio-Rad).

### Serial dilution assays

Mid-log cultures were grown in YPAD or YPAGal liquid media. Five or ten-fold dilutions of the cultures were prepared and plated in solid YPAD or YPAGal plates with the drugs at the indicated concentration. Plates were incubated for 3 days (YPAD) or 5 days (YPAGal) before taking the images shown, except in Supplementary Fig. S2, all of which were incubated for 3 days.

### Re-expression experiments

Exponentially growing cultures were synchronized in G1 with α-factor (0.2 μg/ml) for 3 h in YPARaf or YPAGal medium, then released into MMS 0.05%-containing medium with pronase (50 μg/ml) and nocodazole (5 μg/ml) for 1 h and subsequently treated with 2.5% sodium thiosulphate to inactivate the MMS. The raffinose culture was washed and split in two cultures that were incubated with nocodazole for 2 h in fresh medium without MMS containing either 2% raffinose or 2% galactose. In parallel, the galactose culture was washed and released in fresh galactose medium with nocodazole for 2 h without MMS. In each of the described time points, 100 μl of culture was serially diluted and plated in YPAD medium to measure cell viability. The mean values and standard deviation of the percentage of cell viability resulting from two to five experiments performed are shown.

### BrdU immunofluorescence

The protocol used for immunodetection of BrdU incorporation was adapted from [34]. After DNA labelling and MMS treatment were performed as indicated in each of the experiments, cells were fixed with 4% paraformaldehyde for 20 min at 25°C, washed with water and incubated in 10 mM ditiotreitol (DTT), 0.1 M Na ethylenediaminetetraacetic acid (EDTA; pH 8.0) for 20 min. Then, cells were incubated for 25 min at 25°C with 0.4 mg/ml Zymolyase 20T in 1 M sorbitol, 50 mM sodium phosphate pH 7.0. Spheroplasts were resuspended into the same buffer without zymolyase and 5 μl of the cell suspension were spotted on poly-L-Lysine slides. After 5 min of incubation at 37°C, slides were plunged for 6 min into methanol at −20°C and into acetone for 30 seconds. To allow the detection of incorporated BrdU, fixed slides were denatured with 6 N HCl for 5 min and thoroughly washed with phosphate-buffered saline (PBS) pH 7.0. Slides were incubated for 30 min in blocking buffer containing PBS pH 7.4, 0.1% Tween-20, 1% bovine serum albumin (BSA) (Sigma–Aldrich A4503) prior incubation with primary antibodies. BrdU was detected with 1:20 dilution of rat anti-BrdU clone BU1/75 antibody (Eurobio-AbCys SA, ABC117 7513). Secondary antibody was chicken anti-rat conjugated with Alexa Fluor 488 (Invitrogen, A21470, used at 1:50). For image acquisition, samples were visualized using the 100× oil objective of a Leica DM6000 microscope equipped with a DFC365 FX camera and processed using the LAS AX software (Leica). For signal quantification, Metamorph v7.5.1.0 (Molecular Probes) was used. Box and whiskers (2.5–97.5 percentile) plots show the distribution of nuclear intensities of BrdU signal. The central horizontal line represents the median value. Data are pooled from at least two different experiments with at least 392 cells scored per condition.

### Chromatin fractionation

The protocol used for chromatin fractionation was adapted from [35]. Briefly, 20–30 ml of exponentially growing cultures were blocked with sodium azide 0.1% (w/v) for 15 min on ice, then collected by centrifugation, washed with cold 0.1 mM Tris, pH 9.4, DTT 10 mM and incubated for 15 min in 1 ml of the same buffer on ice. Cells were subsequently washed with cold spheroplast buffer (20 mM HEPES, pH 7.4, 1.2 M sorbitol, Roche Complete EDTA-free protease inhibitor cocktail) and incubated for 1 h at 30°C by adding 0.2 mg/ml Zymolyase 20T in 1 ml of the same buffer. Then, spheroplasts were washed twice with cold wash buffer (20 mM Tris, pH 7.4, 20 mM KCl, 1 M sorbitol, 0.1 μM spermine, 0.25 μM spermidine, Roche Complete EDTA-free protease inhibitor cocktail) and incubated for 10 min on ice with cold lysis buffer (20 mM Tris pH 7.4, 20 mM KCl, 0.4 M sorbitol, 0.1 μM spermine, 0.25 μM spermidine, 1% Triton X-100, Roche Complete EDTA-free protease inhibitor cocktail); 80 μl of the sample were retained as whole cell extract and the remaining sample was centrifugated for 15 min at 15 000 × *g* at 4°C to separate the soluble fraction that remains in the supernatant (160 μl of sample were saved) and the chromatin-enriched pellet. Each pellet was washed with 500 μl of cold lysis buffer, dried, and resuspended in 100 μl of cold water. The amount of protein in each of the total, soluble, and chromatin samples was quantified to obtain a similar protein concentration in all the samples that were then boiled in Laemmli buffer. Soluble and chromatin fractions were analysed by western blot using antibodies against Pgk1 (22C5D8, Invitrogen), histone H4 (ab7311, Abcam), PrimPol [26], and Rfa1 (ab221198, Abcam). All signals were acquired and quantified in a Chemidoc MP imaging system and Image Lab software v6.1.0 (Bio-Rad).

### Electrophoretic mobility shift assay

Electrophoretic mobility shift assay (EMSA) was used to probe that *Hs*PrimPol CH mutant (C419G and H426Y), previously shown to be deficient in primer synthesis [27], could still bind ssDNA. *Hs*PrimPol:ssDNA binding reactions were prepared in buffer E [50 mM Tris-HCl pH 7.5, 40 mM NaCl, 2.5% (w/v) glycerol, 2.5% (w/v) PEG-4000, 1 mM DTT, 0.1 mg/ml BSA]. A PNK-[γ-^32^P]ATP-labeled oligonucleotide (3′-T_20_ GTCCT_36_ 5′, at a concentration of 1 nM) was incubated with increasing concentrations of either wild-type (WT) *Hs*PrimPol or *Hs*PrimPol CH mutant (2.5, 5, 10, 20, 50, 100 nM) in the absence of metal. Reactions (20 μl total volumen) were incubated at 30°C for 10 min. Subsequently, loading buffer (30% glycerol, 1 mM EDTA, 0.1% xylene cyanol and 0.1% bromophenol blue) was added. Samples were analysed on a native 6% polyacrylamide gel which was run at 150 V for 120 min at 4°C in Tris-glycine pH 8.3 buffer. After electrophoresis, the gel was dried and the mobility shift, representing free ssDNA *versus Hs*PrimPol:ssDNA complex, was analysed by autoradiography.

### Structural modelling and bioinformatic analysis

Structural modeling and visualization were performed using UCSF ChimeraX [36]. Multiple sequence alignment was performed using COBALT (NCBI Constraint-based Multiple Alignment Tool; [37]). The sequences used for the alignment were from: *Homo sapiens (NP_002936.1); Equus caballus (XP_023508987.1); Gallus gallus (NP_001006221.1); Columba livia (XP_064892482.1); Chelonia mydas (XP_037736729.1); Dendropsophus ebraccatus (XP_069800696.1); Danio rerio (NP_956105.2); Drosophila melanogaster (NP_524274.1); Rhizopus microspores (XP_023468535.1); Mycosarcoma maydis (AAU05383.1); Zygosaccharomyces parabailii (AQZ17118.1); S. cerevisiae (AJO94941.1)*.

## Results

### Rad53 loss leads to the accumulation of persistent unrepaired lesions

**Figure 1.**
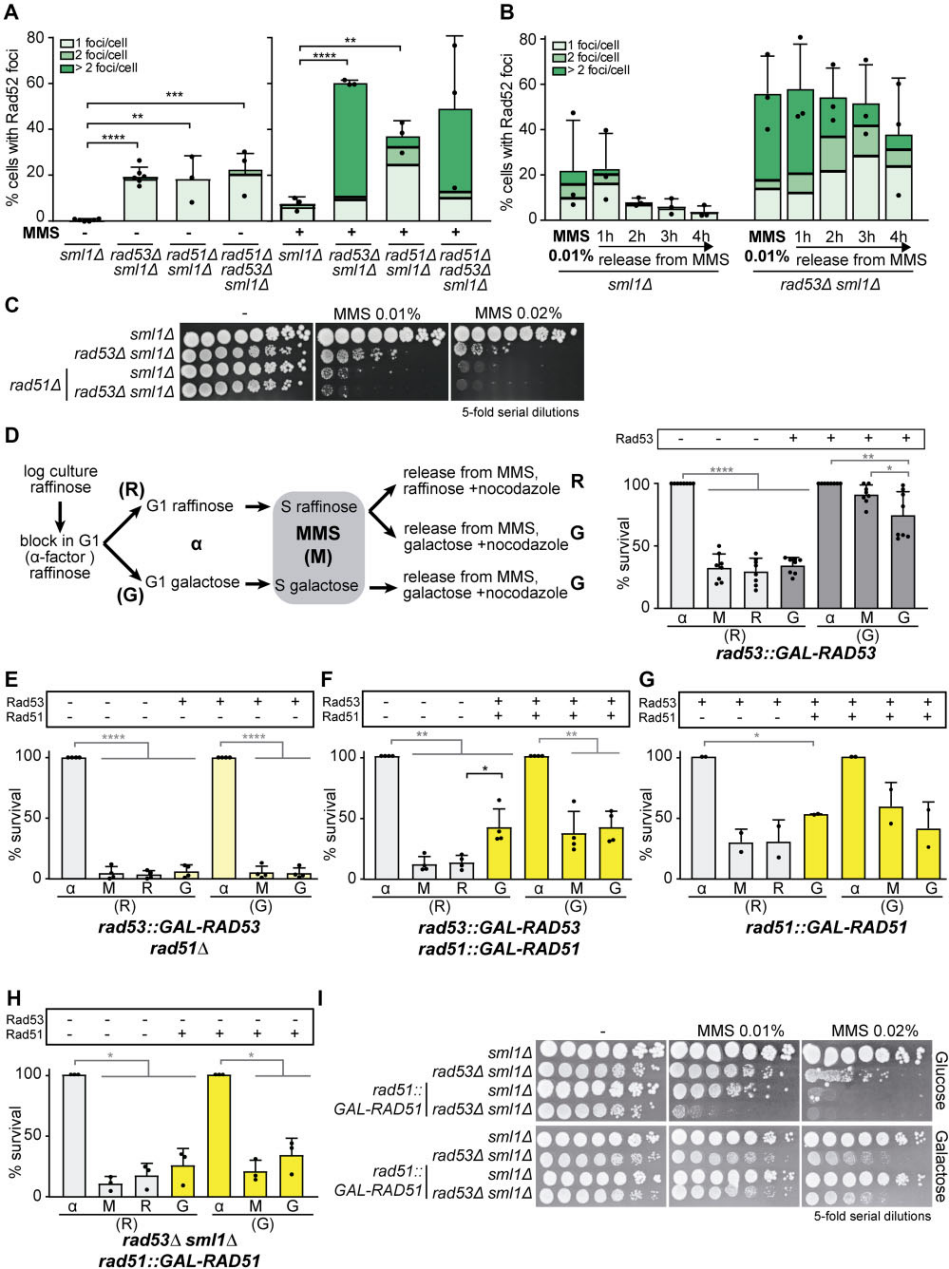
Irreversible fork arrest is due to the aberrant processing of replication forks by Rad51. (**A**) Analysis of the percentage of S/G2 cells with 1, 2, or >2 Rad52-YFP foci in *sml1Δ* (YJT72), *rad53Δ sml1Δ*(YBG437), *rad51Δ sml1Δ* (YLD142), and *rad51Δ rad53Δ sml1Δ* (YBG486) strains transformed with pWJ1344 after release from G1 arrest at 30°C in selective medium for 75 min or in selective medium containing MMS 0.015% for 90 min. (**B**) Analysis of the percentage of S/G2 cells with 1, 2, or >2 Rad52-YFP foci in *sml1Δ* (YJT72), and *rad53Δ sml1Δ* (YBG437) strains transformed with pWJ1344 that were released from G1 arrest at 30°C in selective medium in the presence of the indicated MMS concentration. After 1 h in MMS, cells were treated with 2.5% sodium thiosulphate to inactivate the MMS, washed, and incubated in fresh medium for different times. (**C**) Sensitivity to chronic treatment with different concentrations of MMS of *sml1Δ* (YJT72), *rad53Δ sml1Δ* (YBG437), *rad51Δ sml1Δ* (YLD142), and *rad51Δ rad53Δ sml1Δ* (YBG486) strains, assayed by 5-fold serial dilutions. (**D**) Schematic representation (left panel) of re-expression experiments: α-factor G1 synchronized cells (α) grown in raffinose (R) or galactose (G) were released into S-phase in MMS 0.05% (M) and nocodazole-containing medium for 1 h. The raffinose culture (R) was then divided in two cultures that were incubated for 2 h in fresh medium without MMS containing either raffinose (R) or galactose (G) and nocodazole. In parallel, the galactose culture (G) was released in fresh galactose and nocodazole medium for 2 h without MMS as checkpoint-proficient control. Cell viability (right panel) measured during the re-expression experiment performed with *rad53::GAL-RAD53* (YIN001) strain (n = 8). (**E**–**H**) Cell viability measured during re-expression experiments performed with *rad53::GAL-RAD53 rad51Δ* (YBG572) (E), *rad53::GAL-RAD53 rad51::GAL-RAD51* (YBG571) (F), *rad51::GAL-RAD51* (YBG573) (G), and *rad53Δ sml1Δ rad51::GAL-RAD51* (YIN139) (H) strains (n ≥ 2). Other details as in Fig. 1D. (**I**) Sensitivity to chronic treatment with different concentrations of MMS of *sml1Δ* (YBG621), *rad53Δ sml1Δ* (YBG501), *sml1Δ rad51::GAL-RAD51* (YIN138), and *rad53Δ sml1Δ rad51::GAL-RAD51*(YIN139) strains, assayed by 5-fold serial dilutions. In panels (A) and (B), a minimum of 100 cells were counted per experiment. Each dot represents the percentage of cells with Rad52 foci out of the total number of cells in each experiment. Mean and standard deviation (SD) from at least three experiments (n ≥ 3) are shown. **P < .01, ***P < .001, ****P < .0001 (two-tailed unpaired *t*-test). In panels (D), (E), (F), (G), and (H) each dot represents the percentage of survival for each time point per experiment. Mean and SD from at least two experiments (n ≥ 2) are shown. *P < .05, **P < .01, ****P < .0001 (two-tailed paired *t*-test). Black stars denote significant increases, whereas grey stars denote significant decreases.

Upon DNA damage, the S-phase checkpoint could be involved in reducing the occurrence of DNA lesions, in ensuring their proper repair, or in both. Thus, the high number of Rad52 foci reported in checkpoint mutants could be caused by either a higher frequency of DNA lesions, or from defective repair mechanisms. To distinguish between these possibilities, we examined the effects of Rad51 loss, which functions downstream of Rad52 in double-strand break (DSB) repair. We hypothesized that if checkpoint mutants led to an increase in DNA lesions, the absence of Rad51 (*rad51Δ*) would exacerbate the accumulation of Rad52 foci due to impaired repair. On the other hand, if checkpoint mutants were already experiencing defective repair, the *rad51Δ* mutation would be epistatic, and we would not expect a further increase in Rad52 foci. We conducted these experiments in the *sml1Δ* background to allow the survival of *rad53Δ* cells. In untreated cells, *rad51Δ* caused a 20-fold increase in the frequency of Rad52 foci (Fig. 1A), consistent with previous observations [38] and with the accumulation of unrepaired spontaneous DNA lesions. Notably, in the double mutant *rad51Δ rad53Δ*, the percentage of cells with Rad52 foci did not increase beyond that seen in the single mutants, suggesting that lesions that arise spontaneously during S phase, which would normally be repaired by Rad51, are left unrepaired and persist at the stage in which Rad52 is loaded. A similar pattern was observed under replicative stress, as seen in cells treated with MMS. While MMS treatment increased the percentage of cells with Rad52 foci in all strains, no additional increase was observed in the double mutant *rad51Δ rad53Δ* compared to the single mutants. These findings indicate that Rad52 foci accumulation is primarily a consequence of defective lesion repair.

To further validate this, we assessed the recovery of cells after a 1-h MMS treatment. As shown in Fig. 1B, the percentage of *sml1Δ* cells with Rad52 foci decreased from 20% to <5% within 4 h, suggesting that ∼75% of the foci were repaired in checkpoint-proficient conditions. In contrast, >50% of *rad53Δ sml1Δ* cells exhibited Rad52 foci after MMS treatment, and only 20% of these foci were repaired within 4 h. These results indicate that the loss of Rad53 leads to the accumulation of persistent, unrepaired lesions, and the high levels of Rad52 foci observed in *rad53Δ* cells is due to defective repair of stalled replication forks caused by both endogenous and exogenous DNA damage. Thus, Rad52 foci can reflect either accurate processing of spontaneous lesions that would lead to repair, as they appear in WT cells, or aberrant processing, as they accumulate in checkpoint-deficient cells.

### Irreversible replication fork arrest depends on Rad51

Our observations suggest that the high sensitivity to DNA damage of checkpoint mutants may result from aberrant processing of arrested replication forks by Rad51, which can be detected as Rad52 foci. Therefore, Rad51 (and Rad52) would be involved in DNA damage sensitivity of *rad53Δ* cells. However, *rad51Δ* did not rescue the viability of *rad53Δ* cells after chronic MMS exposure as shown by serial dilution assays even at low MMS doses, in which the survival of the double *rad53Δ rad51Δ* mutant was lower than that of the single *rad51Δ* or *rad53Δ* mutants (Fig. 1C). These results align with previous findings in *rad52Δ* cells [21] and are likely explained by the extreme MMS sensitivity of the *rad51Δ* and *rad52Δ* single mutants. Thus, we next explored the role of Rad51 in fork arrest by studying the effect of its specific loss during the MMS treatment.

Irreversible fork arrest was initially defined in re-expression experiments, where the survival of checkpoint-deficient cells following HU treatment could not be restored, even after checkpoint factors were re-expressed post-HU removal, in a strain in which the sole copy of *RAD53* was under the *GAL* promoter (*rad53::GAL-RAD53*) [14]. Since later experiments have shown that survival after HU treatment requires additional checkpoint-dependent ribonucleotide reductase induction [39], we used MMS to investigate the potential role of Rad51 in fork arrest through these re-expression experiments (Fig. 1D and Supplementary Fig. S1A). Cells were synchronized with α-factor and released into S phase in raffinose-containing medium (R), where Rad53 is not expressed. After treatment with MMS, these cells showed a significant loss of viability, dropping to <30%, as expected. Releasing these cells from MMS into galactose-containing medium, which induces *RAD53* expression, did not restore viability, indicating that the effects of MMS in *rad53Δ* cells are irreversible and consistent with previous findings for HU [14]. As a checkpoint-proficient control, the entire experiment was also conducted in galactose-containing medium (G), where Rad53 is continuously expressed. Under these conditions, MMS exposure did not result in a significant loss of viability, although a moderate decrease was observed after MMS removal, likely due to Rad53 overexpression driven by the *GAL* promoter, as reported previously with HU [14].

Further re-expression experiments revealed that complete loss of Rad51 (*rad51Δ*) caused severe lethality upon MMS treatment, and viability could not be restored upon release from MMS, even in the checkpoint-proficient condition in galactose medium (G) (Fig. 1E and Supplementary Fig. S1B). However, given the reported role of Rad51 in fork resumption or progression even in the absence of DNA damage [40–42], we investigated the role of Rad51 in fork arrest with a strain where the only copy of each of *RAD53* and *RAD51* genes is regulated by the *GAL* promoter (*rad53::GAL-RAD53 rad51::GAL-RAD51*) so that not only Rad53, but also Rad51 expression, could be controlled during and after MMS treatment and removal (Fig. 1F and Supplementary Fig. S1C). Notably, release from MMS into galactose-containing medium, to induce the expression of both *RAD51* and *RAD53*, restored survival from 20% to 40%, which is the same level as the control condition in which both factors were expressed during the whole experiment in agreement with the reported toxic effect of *RAD51* overexpression [43]. We confirmed that the MMS-induced effect was not irreversible upon Rad51 loss in a checkpoint-proficient strain (Fig. 1G and Supplementary Fig. S1D). Furthermore, neither the lack of *RAD51* expression nor its overexpression could increase the viability of *rad53Δ* cells in re-expression experiments or during chronic MMS treatment (Fig. 1H and I). Taken together, these results suggest that both the loss of Rad51 during MMS treatment and its expression together with Rad53 after MMS removal are essential for cell viability.

In summary, our data support the idea that irreversible replication fork arrest is mediated by Rad51, although this role has not previously been recognized, likely because it also participates in fork progression or resumption. Re-expression experiments provide new insights to distinguish the effect of Rad51 loss on fork arrest from its role in fork progression or resumption once the damaging agent is removed. Therefore, this system of controlling expression of particular genes in a single cell cycle offers a novel approach to identify factors and domains involved in fork arrest.

### Identification of Mre11, Mus81, and Rad5 as potential factors required for irreversible fork arrest

To investigate which other repair factors contribute to irreversible replication fork arrest, we conducted a targeted screen of potential candidates. We selected those nonessential factors whose overexpression is viable and, similar to Rad51, are involved in lesion repair and form foci in S phase with or without replication stress. Such factors included nucleases Mre11, Sae2, and Mus81 [44] and helicases Sgs1 [13], Srs2 [45], Rad5 [46], Rad54 [13], and Mph1-Mte1 [47]. We also included the nonessential Pol ζ subunit Pol32 that is involved in the repair of one-ended DNA DSBs, such as those originated at broken replication forks, and the Shu1 subunit of the Shu complex, which promotes recombinational repair at replication forks [48].

We generated strains where the sole copies of both *RAD53* and the respective candidate genes were placed under the control of the *GAL* promoter. All such strains grew well in glucose or galactose media (Supplementary Fig. S2A), except in the cases of Mre11 and Sgs1, in agreement with their reported genetic interactions with Rad53 [49]. In galactose media (checkpoint-proficient conditions), MMS sensitivity was only observed upon *SRS*2 overexpression, likely related with its reported toxicity [50, 51]. In glucose media, in contrast and as expected from the repression of Rad53 expression, high MMS concentrations severely impaired growth in all cases. Some strains were already sensitive even at lower doses, in agreement with the reported strong MMS sensitivity of the single mutants or their negative genetic interactions with Rad53 [49].

**Figure 2.**
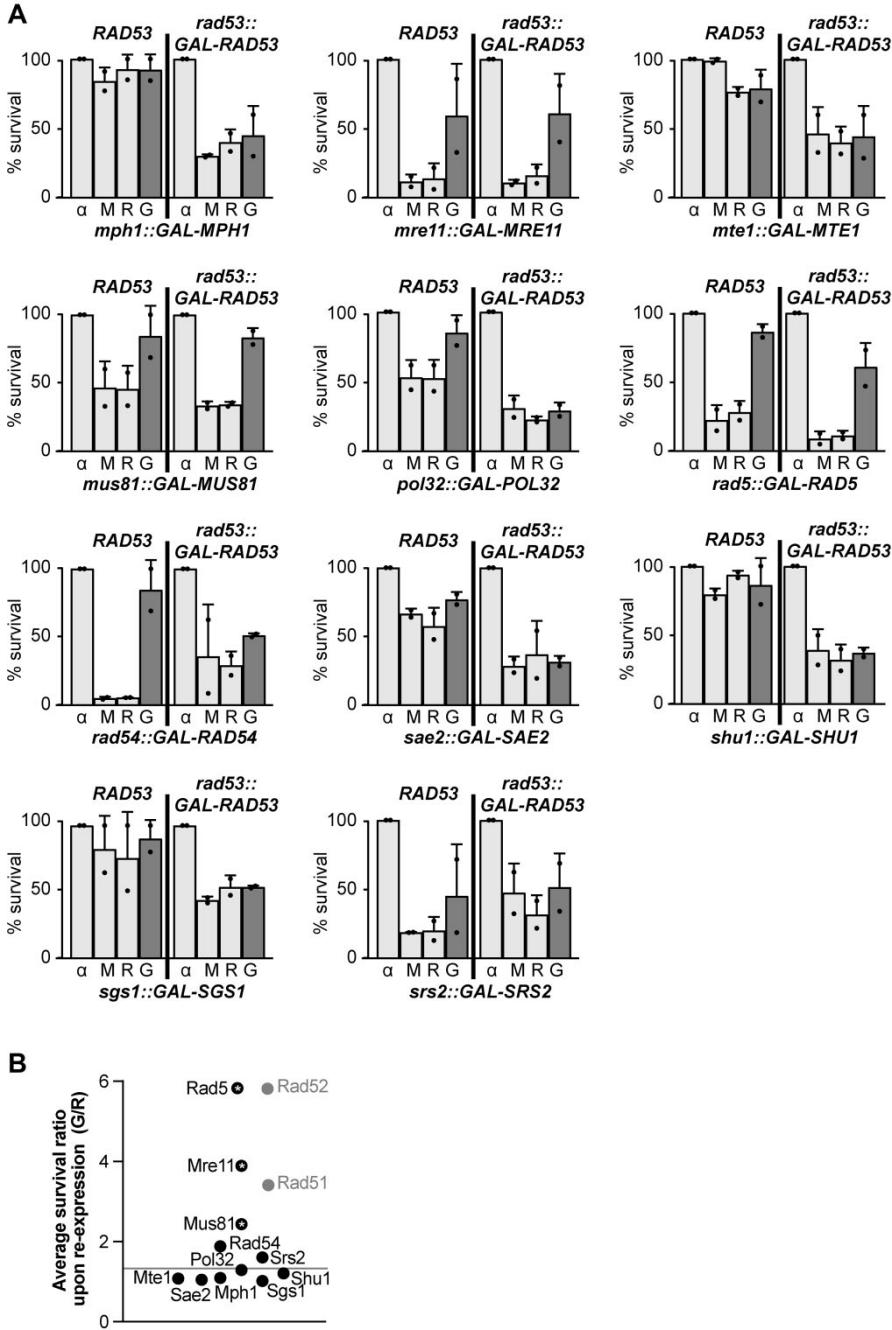
Role of different repair factors in irreversible fork arrest. (**A**) Cell viability measured during re-expression experiments. Other details as in Fig. 1D. In each panel, the left part of each graph represents the re-expression experiment with the control checkpoint-proficient strains in which only the sole copy of each of the selected gene is under the regulation of *GAL* promoter, while the right part of each graph corresponds to the strain with both Rad53 and the selected gene under *GAL* promoter. The strains used were *mph1::GAL-MPH1* (YIN016), *rad53::GAL-RAD53 mph1::GAL-MPH1* (YIN018), *mre11:GAL-MRE11* (YIN020), *rad53::GAL-RAD53 mre11::GAL-MRE11* (YIN021), *mte1::GAL-MTE1* (YIN043), *rad53::GAL-RAD53 mte1::GAL-MTE1* (YIN060), *mus81::GAL-MUS81* (YIN003), *rad53::GAL-RAD53 mus81::GAL-MUS81* (YIN005), *pol32::GAL-POL32* (YIN023), *rad53::GAL-RAD53 pol32::GAL-POL32* (YIN025), *rad5::GAL-RAD5* (YIN141), *rad53::GAL-RAD53 rad5::GAL-RAD5* (YIN112), *rad54::GAL-RAD54* (YIN026), *rad53::GAL-RAD53 rad54::GAL-RAD54* (YIN028), *sae2::GAL-SAE2* (YIN049), *rad53::GAL-RAD53 sae2::GAL-SAE2* (YIN056), *shu1::GAL-SHU1* (YIN039), *rad53::GAL-RAD53 shu1::GAL-SHU1* (YIN058), *sgs1::GAL-SGS1* (YIN008), *rad53::GAL-RAD53 sgs1::GAL-SGS1* (YIN011), *srs2::GAL-SRS2* (YIN035), and *rad53::GAL-RAD53 srs2::GAL-SRS2* (YIN062). Each dot of the graphs represents the percentage of survival for each time point per experiment. Mean and SD from two experiments (n = 2) are shown. (**B**) Average survival ratio upon re-expression. The percentage of survival when, after MMS removal, each of the indicated factors were re-expressed in galactose (G) versus when they were repressed in raffinose (R) in the *rad53::GAL-RAD53* strain background calculated from at least two experiments. *P< .05 (one sample *t*-test versus the average level of the parental *rad53::GAL-RAD53* strain, 1.31, which is represented as an horizontal line).

Even when the loss of the single factors caused MMS sensitivity, re-expression experiments revealed that it was reversible in the presence of Rad53 (Fig. 2A, left panels). In contrast, lethality persisted in the *rad53::GAL-RAD53* background in most strains (Fig. 2A, right panels). This is consistent with the notion that replication fork arrest is irreversible in checkpoint-deficient cells even when *RAD53* expression is restored after MMS removal. Strikingly, we observed a partial rescue of viability when Mre11, Mus81, or Rad5 were absent during MMS treatment and overexpressed, in addition to Rad53, after MMS removal. In a similar manner to Rad51 or Rad52, the ratio of survival after re-expression (G/R) in these three cases was significantly higher as compared to the average ratio of survival after re-expression of the parental *rad53::GAL-RAD53* strain (Fig. 2B). These results suggest that such factors may be involved in irreversible fork arrest and, like Rad51, were not identified previously because they participate in fork progression or resumption after MMS removal. To evaluate this possibility, we used a genetic background optimized for BrdU incorporation [52], and the ability to incorporate BrdU after a pulse of 30 min of MMS treatment was quantitatively assessed by immunofluorescence (Supplementary Fig. S2B). As expected from the irreversible arrest of ongoing forks, *rad53Δ sml1Δ* cells showed a significant reduction in the BrdU signal intensity after MMS, and this was not recovered by *rad51Δ* or by *mre11Δ, mus81Δ*, or *rad5Δ* mutants. Thus, we decided to focus on these three candidates to be involved in replication fork arrest, together with Rad51.

### Irreversible fork arrest can be suppressed by *MRE11* overexpression independently of its catalytic activity

**Figure 3.**
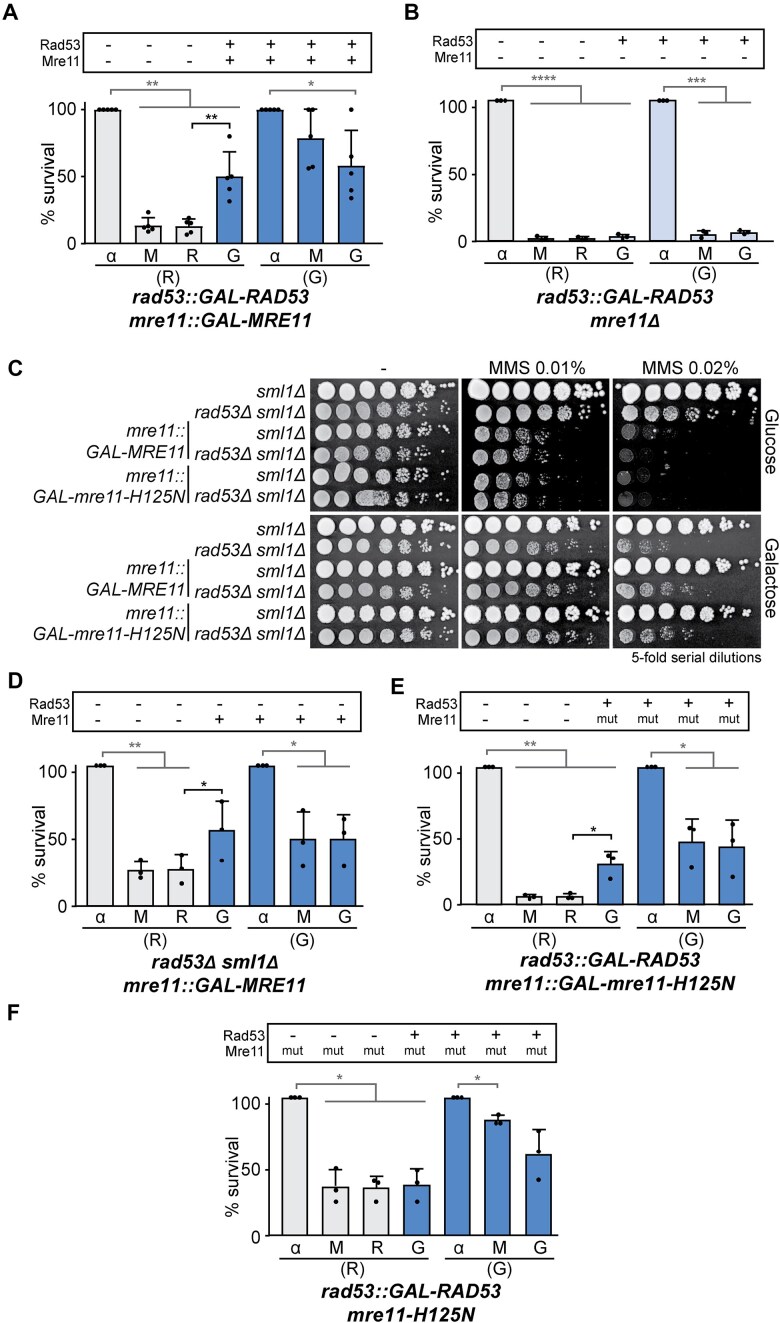
Irreversible fork arrest can be suppressed by *MRE11* overexpression, independently on its catalytic activity. (**A**, **B**, **D**, **E**, **F**) Cell viability measured during re-expression experiments performed with *rad53::GAL-RAD53 mre11::GAL-MRE11* (YIN021) (**A**), *rad53::GAL-RAD53 mre11Δ* (YIN077) (**B**), *rad53Δ sml1Δ mre11::GAL-MRE11* (YIN047) (**D**), *rad53::GAL-RAD53 mre11::GAL-mre11-H125N* (YIN081) (**E**), and *rad53::GAL-RAD53 mre11-H125N* (YIN140) (**F**) strains. Other details as in Fig. 1D. (**C**) Sensitivity to chronic treatment with different concentrations of MMS of *sml1Δ* (YBG621), *rad53Δ sml1Δ* (YBG501), *sml1Δ mre11::GAL-MRE11* (YIN053), *rad53Δ sml1Δ mre11::GAL-MRE11* (YIN047), *sml1Δ mre11::GAL-mre11-H125N* (YIN109), and *rad53Δ sml1Δ mre11::GAL-mre11-H125N* (YIN111) strains, assayed by 5-fold serial dilutions. In panels (A), (B), (D), (E), and (F), each dot represents the percentage of survival for each time point per experiment. Mean and SD from at least three experiments (n ≥ 3) are shown. *P < .05, **P < .01, ***P < .001, ****P < .0001 (two-tailed paired *t*-test). Black stars denote significant increases, whereas grey stars denote significant decreases.

Mre11, the nuclease subunit of the MRX complex (comprising Mre11, Rad50, and Xrs2), is known to function in DSB repair and post-replication repair processes [53–55]. To validate its potential role in replication fork arrest, we performed re-expression experiments, including the checkpoint-proficient control using galactose (G) medium in which we confirmed that *MRE11* overexpression does not further affect MMS survival (Fig. 3A). In raffinose (R), however, we observed a significant rescue of cell viability specifically when both *RAD53* and *MRE11* expression were repressed during MMS treatment and subsequently expressed after MMS removal. A similar rescue was observed in re-expression experiments with *RAD50*, but not with *XRS*2 (Supplementary Fig. S3A and B). In contrast, deletion of the *MRE11* gene did not rescue viability but instead exacerbated the loss of survival following MMS treatment, even in the presence of Rad53 (galactose medium) (Fig. 3B). Thus, the loss and re-expression of *MRE11* or *RAD50* can overcome MMS-induced arrest of checkpoint deficient cells. To examine whether this rescue observed by *MRE11* re-expression requires other MRX subunits, we conducted re-expression experiments in *rad50Δ* and *xrs2Δ*. However, given the strong sensitivity caused by MRX loss, we observed almost no MMS survival in these cases, even in the presence of the checkpoint (galactose) (Supplementary Fig. S3C and D).

To test if the rescue of cell viability resulted from the absence of Mre11 during the MMS treatment or from *MRE11* overexpression itself, we assessed survival under chronic MMS exposure by serial dilutions of *rad53Δ sml1Δ mre11::GAL-MRE11* cells. Lack of Mre11 (glucose medium) increased MMS sensitivity as anticipated, whereas *MRE11* overexpression (galactose medium) modestly improved MMS survival in *rad53Δ* cells (Fig. 3C). Similar results were obtained in re-expression experiments using this strain (Fig. 3D), where the decrease in viability was reversed without requiring *RAD53* re-expression after damage. Moreover, a slight improvement of the survival to high MMS concentrations was also observed upon overexpression of *RAD50*, but not of *XRS2* under chronic MMS exposure (Supplementary Fig. S3E). These results suggest that the observed viability rescue may be driven by *MRE11* or *RAD50* overexpression rather than their absence during DNA damage.

Next, we examined whether the nuclease activity of Mre11 was necessary for this rescue by overexpressing the *mre11-H125N* mutant, which lacks nuclease activity but retains the ability to form the MRX complex [53, 56]. Chronic MMS exposure (Fig. 3C) and re-expression experiments (Fig. 3E) revealed similar viability rescue effects with both WT *MRE11* and the nuclease-deficient *mre11-H125N* mutant overexpression. However, expressing *mre11-H125N* at endogenous levels in a *rad53::GAL-RAD53* background did not alleviate irreversible fork arrest (Fig. 3F). Thus, the lack of Mre11 catalytic activity during the MMS treatment has no effect on survival of checkpoint-deficient cells and the viability rescue appears to be mediated by an overabundance of the Mre11 protein, independently of its catalytic activity.

In summary, overexpression of either WT *MRE11, RAD50*, or the *mre11-H125N* mutant, can mitigate the toxicity of MMS that leads to fork arrest.

### Irreversible fork arrest is dependent on Mus81 catalytic activity

**Figure 4.**
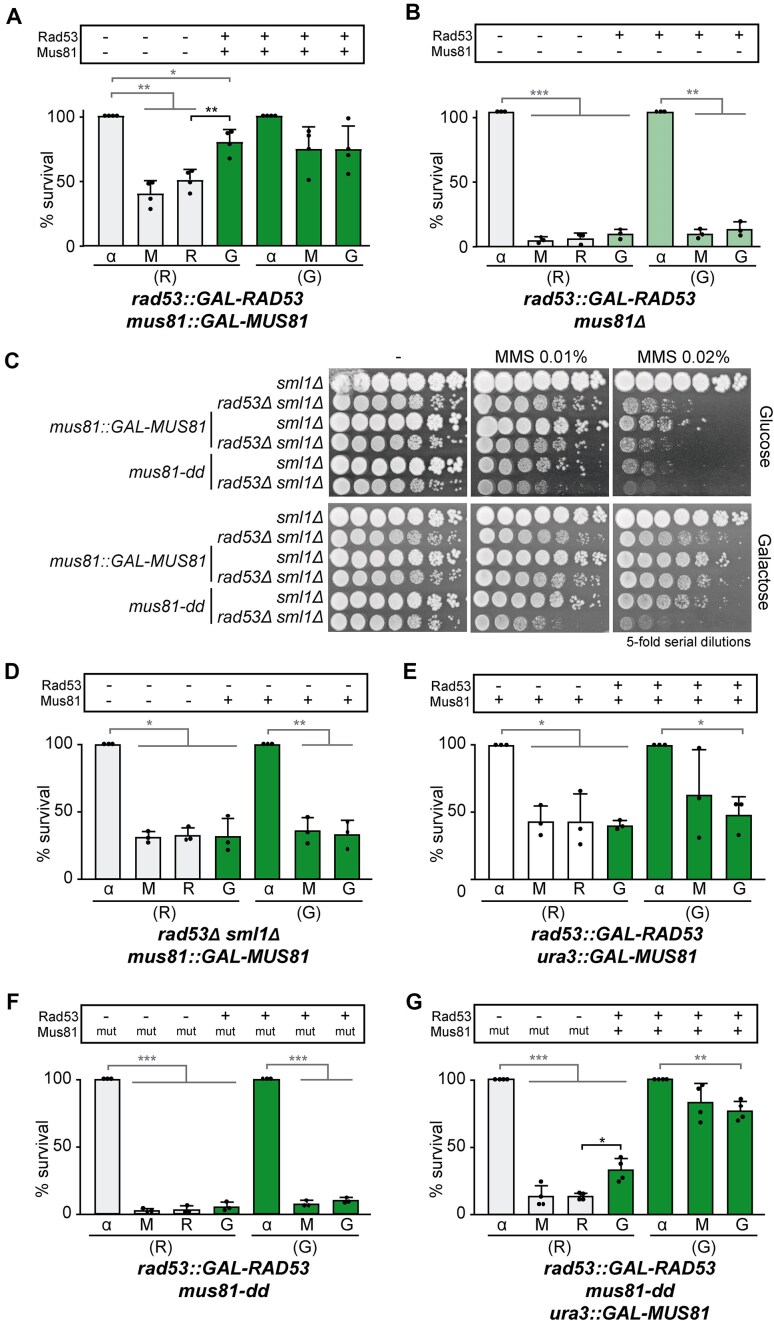
Irreversible fork arrest is dependent on Mus81 catalytic activity. (**A**, **B**, **D**, **E**, **F**, **G**) Cell viability measured during re-expression experiments performed with *rad53::GAL-RAD53 mus81::GAL-MUS81* (YIN005) (**A**), *rad53::GAL-RAD53 mus81Δ* (YIN074) (**B**), *rad53Δ sml1Δ mus81::GAL-MUS81* (YIN030) (**D**), *rad53::GAL-RAD53 ura3::GAL-MUS81* (YIN085) (**E**), *rad53::GAL-RAD53 mus81-dd* (YIN083) (**F**), and *rad53::GAL-RAD53 mus81-dd ura3::GAL-MUS81* (YIN088) (**G**) strains. Other details as in Fig. 1D. (**C**) Sensitivity to chronic treatment with different concentrations of MMS of *sml1Δ* (YBG621), *rad53Δ sml1Δ* (YBG501), *sml1Δ mus81::GAL-MUS81* (YIN135), *rad53Δ sml1Δ mus81::GAL-MUS81* (YIN030), *sml1Δ mus81-dd* (YIN142), and *rad53Δ sml1Δ mus81-dd* (YIN143) strains, assayed by 5-fold serial dilutions. In panels (A), (B), (D), (E), (F), and (G), each dot represents the percentage of survival for each time point per experiment. Mean and SD from at least three experiments (n ≥ 3) are shown. *P < .05, **P < .01, ***P < .001 (two-tailed paired *t*-test). Black stars denote significant increases, whereas grey stars denote significant decreases.

Mus81, the nuclease subunit of the Mms4-Mus81 structure-specific endonuclease, is implicated in processing stalled replication forks and DSB repair [57–60]. To confirm its potential involvement in fork arrest, we performed re-expression experiments in the *rad53::GAL-RAD53 mus81::GAL-MUS81* strain. A marked rescue of viability was observed when, in addition to *RAD53*, *MUS81* was repressed during MMS treatment and subsequently overexpressed after MMS removal (Fig. 4A). However, this effect was not observed when Mus81 was absent throughout experiment (Fig. 4B). Importantly, and in contrast to what we observed for *MRE11*, overexpression of *MUS81* alone was insufficient to rescue MMS-induced toxicity in *rad53Δ sml1Δ* cells, as shown by serial dilution assays under chronic MMS exposure (Fig. 4C) or by re-expression experiments (Fig. 4D). Cell viability was not rescued either when endogenous *MUS81* expression was allowed during MMS treatment in addition to its overexpression after MMS removal (Fig. 4E). These findings indicate that *MUS81* overexpression alone cannot overcome fork arrest when *MUS81* is present during the MMS treatment. In contrast, its loss and the expression of both Rad53 and Mus81 after MMS removal are required to restore cell viability.

To assess whether the nuclease activity of Mus81 is critical for irreversible fork arrest, we investigated the effect of the nuclease-dead mutant *mus81-dd* (*mus81-D414A, D415A*) [61]. This mutant significantly reduced survival after MMS treatment, even in the presence of Rad53 (galactose medium) (Fig. 4C and F). This finding suggests that the nuclease activity of Mus81 is essential for survival after MMS removal, consistent with its reported role in fork resumption [62]. Therefore, we performed the re-expression experiments with an additional WT copy of *MUS81* that was only expressed after MMS removal (Fig. 4G). In this strain, we observed a significant rescue of cell viability, indicating that the loss of Mus81 catalytic activity during MMS treatment was sufficient to allow recovery of viability under DNA damage conditions in checkpoint-deficient cells. Thus, these results support that irreversible replication fork arrest depends on the catalytic activity of Mus81.

### Irreversible fork arrest is dependent on Rad5 helicase and HIRAN domains but not on its ubiquitin ligase activity

**Figure 5.**
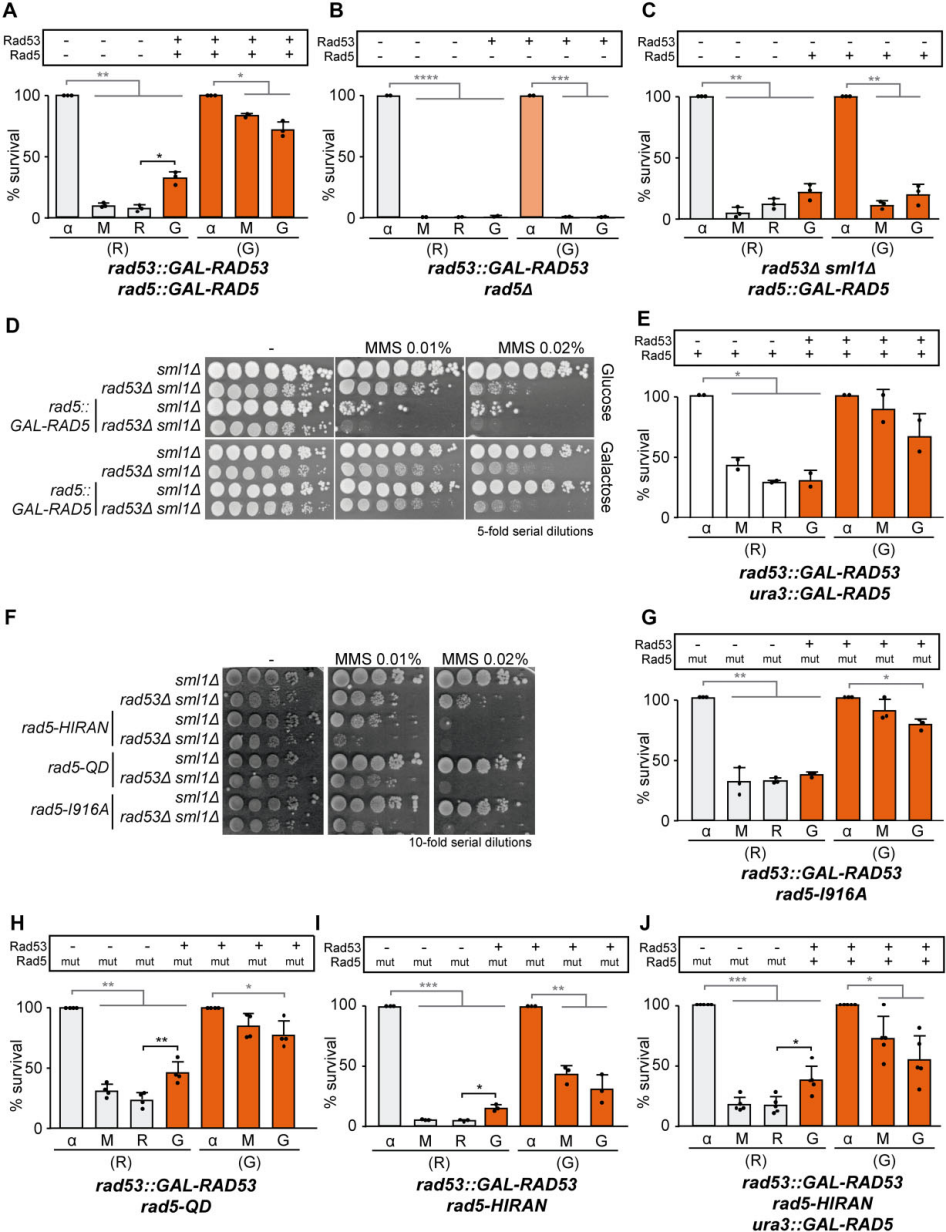
Irreversible fork arrest is dependent on Rad5 helicase and HIRAN domains but not on its ubiquitin ligase activity. (**A**, **B**, **C**, **E**, **G**, **H**, **I**, **J**) Cell viability measured during re-expression experiments performed with *rad53::GAL-RAD53 rad5::GAL-RAD5* (YIN112) (**A**), *rad53::GAL-RAD53 rad5Δ* (YBG592) (**B**), *rad53Δ sml1Δ rad5::GAL-RAD5* (YIN137) (**C**), *rad53::GAL-RAD53 ura3::GAL-RAD5* (YIN089) (**E**), *rad53::GAL-RAD53 rad5-I916A* (YIN093) (**G**), *rad53::GAL-RAD53 rad5-QD* (YIN094) (**H**), *rad53::GAL-RAD53 rad5-HIRAN* (YIN080) (**I**), and *rad53::GAL-RAD53 rad5-HIRAN ura3::GAL-RAD5* (YIN091) (**J**) strains. Other details as in Fig. 1D. (**D**) Sensitivity to chronic treatment with different concentrations of MMS of *sml1Δ* (YBG621), *rad53Δ sml1Δ* (YBG501), *sml1Δ rad5::GAL-RAD5* (YIN136), and *rad53Δ sml1Δ rad5::GAL-RAD5* (YIN137) strains, assayed by 5-fold serial dilutions. (**F**) Sensitivity to chronic treatment with different concentrations of MMS of *sml1Δ* (YBG621), *rad53Δ sml1Δ* (YBG501), *sml1Δ rad5-HIRAN* (YIN104), rad53Δ *sml1Δ rad5-HIRAN* (YIN105), *sml1Δ rad5-QD* (YIN146), rad53Δ *sml1Δ rad5-QD* (YIN147), *sml1Δ rad5-I916A* (YIN107), and *rad53Δ sml1Δ rad5-I916A* (YIN108) strains, assayed by 10-fold serial dilutions. In panels (A), (B), (C), (E), (G), (H), (I), and (J), each dot represents the percentage of survival for each time point per experiment. Mean and SD from at least two experiments (n ≥ 2) are shown. *P < .05, **P < .01, ***P < .001, ****P < .0001 (two-tailed paired *t*-test). Black stars denote significant increases, whereas grey stars denote significant decreases.

Re-expression experiments in the *rad53::GAL-RAD53 rad5::GAL-RAD5* strain confirmed a significant rescue of cell viability when Rad5 expression was allowed only after MMS treatment and removal (R) and no major viability loss was detected in galactose control conditions (G) (Fig. 5A). However, this rescue was not observed in *rad53::GAL-RAD53 rad5Δ* (Fig. 5B) or in *rad53Δ sml1Δ rad5::GAL-RAD5* strains, either in re-expression experiments (Fig. 5C) or during chronic exposure to MMS (Fig. 5D). In addition, allowing endogenous *RAD5* expression during the MMS treatment and inducing its overexpression only after MMS removal was unable to rescue by itself (Fig. 5E). Thus, Rad5 overexpression is not enough and both the loss of Rad5 and the re-expression of Rad53 and Rad5 activities are essential after MMS removal to enable recovery of cell survival.

Rad5 has several enzymatic activities [63–68]. Both Rad5 and its human orthologue HLTF share a HIP116 Rad5p N-terminal (HIRAN) domain and a SWI2/SNF2 helicase-homologous region, which confer DNA-dependent ATPase and helicase activities. Additionally, they contain a RING finger domain responsible for catalysing PCNA poly-ubiquitination, which is of importance in post-replicative repair. To investigate the roles of the distinct domains of Rad5, we analysed the effects of three previously characterized mutants: *rad5-I916A*, which is defective in PCNA poly-ubiquitination but retains helicase activity [69]; *rad5-QD*, which is helicase-deficient but unaffected in PCNA poly-ubiquitination [70]; and *rad5-HIRAN (R187E)*, which disrupts template switching reactions such as fork reversal [71]. Under checkpoint-proficient conditions (*sml1Δ*), only the *rad5-HIRAN* mutant exhibited increased MMS sensitivity (Fig. 5F), suggesting that this domain may play a role in fork resumption. Additionally, combining the *rad5* point mutants with *rad53Δ* significantly increased MMS sensitivity in all cases (Fig. 5F), indicating that none of the mutations alone could compensate for the loss of RAD53 activity. In re-expression experiments, we observed that the *rad5-I916A* did not affect the survival of *rad53::GAL-RAD53* cells (Fig. 5G). In contrast, *rad5-QD* led to a significant rescue of viability (Fig. 5H), suggesting that replication fork arrest is mediated by the helicase activity of Rad5 rather than its ubiquitin ligase function. The *rad5-HIRAN* mutation provided a modest rescue of MMS-induced viability loss in *rad53::GAL-RAD53* after MMS treatment (Fig. 5I). Notably, this strain displayed impaired growth under galactose control conditions, indicating that the HIRAN domain is essential for cell viability following MMS removal in agreement with the previous conclusion that it may play a role in fork resumption. To address this issue, we introduced a WT copy of *RAD5* under the *GAL* promoter allowing its expression to be induced after MMS treatment (Fig. 5J). In this case, cell survival was significantly restored, indicating that the absence of the HIRAN domain during MMS treatment was sufficient to promote survival if its activity is allowed following MMS removal. Collectively, these findings indicate that irreversible replication fork arrest relies on the helicase and HIRAN domains of Rad5, while its ubiquitin ligase activity is dispensable for this process.

### Lack of Rad53 results in nascent DNA degradation dependent on Rad51, Rad5, and Mus81, but not on Mre11

**Figure 6.**
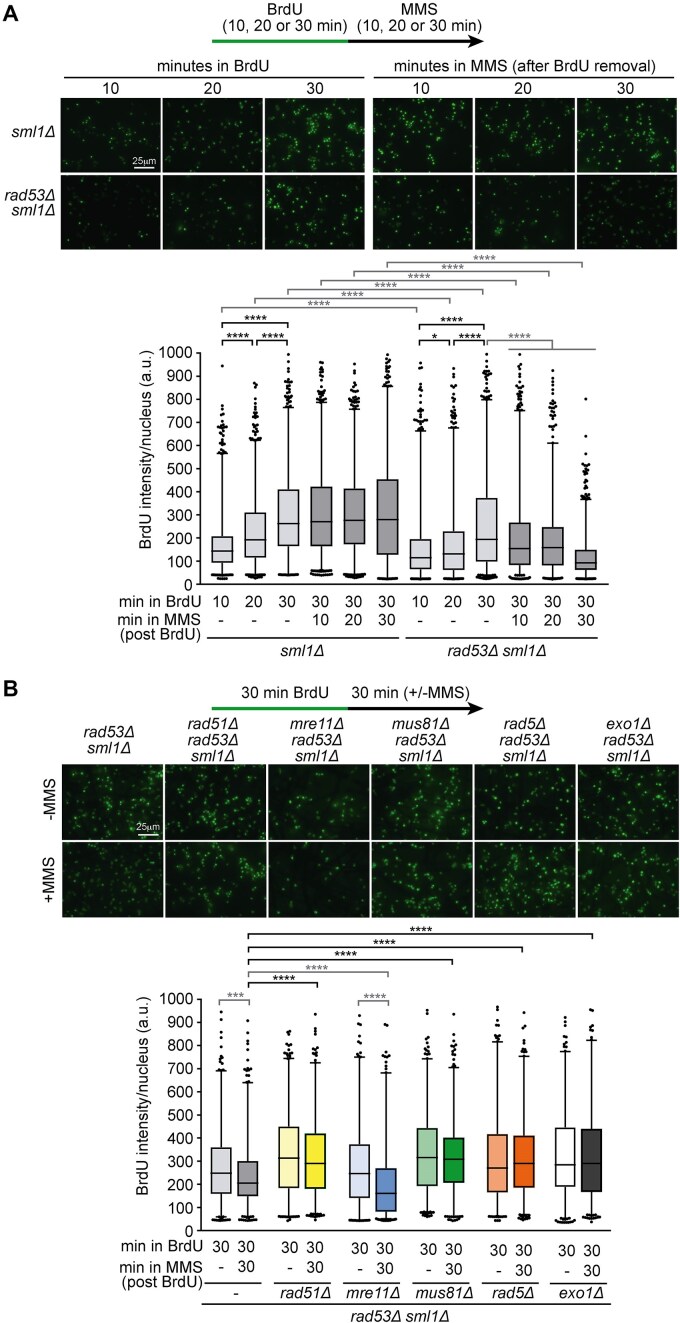
Nascent DNA degradation requires Rad51, Rad5, and Mus81, but not Mre11. (**A**) Schematic of the experimental set-up (top panel): cells were labelled with 200 μM BrdU for 10, 20, or 30 min, washed and released in fresh YPAD medium without BrdU for 30 min, and then treated for immunofluorescence as described in Materials and Methods. In parallel, cultures labelled with BrdU for 30 min were released in fresh YPAD medium without BrdU and treated with MMS 0.02% for 10, 20, or 30 min before being processed for immunofluorescence. Immunodetection (bottom panel) of the intensity of BrdU incorporated in asynchronously growing cultures of *sml1Δ* (YIN122) and *rad53Δ sml1Δ* (YIN124) strains. Representative images of the different conditions are shown. Scale bar, 25 μm. (**B**) Schematic of the experimental set-up (top panel): cells were pulse-labelled with 200 μM BrdU for 30 min and then washed and released in fresh YPAD medium without BrdU and with or without MMS 0.02%. Immunodetection (bottom panel) of the intensity of BrdU incorporated in asynchronously growing cultures of *rad53Δ sml1Δ* (YIN124), *rad53Δ sml1Δ rad51Δ* (YIN174), *rad53Δ sml1Δ mre11Δ* (YIN129), *rad53Δ sml1Δ mus81Δ* (YIN126), *rad53Δ sml1Δ rad5Δ* (YIN130), and *rad53Δ sml1Δ exo1Δ* (YIN128) strains. Representative images of the different conditions are shown. Scale bar, 25 μm. In panels (A) and (B), box and whiskers (2.5–97.5 percentile) plots show the distribution of BrdU intensities per yeast nuclei. The central horizontal line represents the median value. A minimum of 606 cells per condition from three independent experiments were plotted (n > 606). *P < .05, ***P < .001, ****P < .0001 (two-tailed Mann–Whitney U test). Black stars denote significant increases, whereas grey stars denote significant decreases.

Given the critical role of Mus81 nucleolytic cleavage in irreversible fork arrest, we investigated whether aberrant fork processing involves the degradation of nascent DNA. To measure nascent DNA integrity, cells were treated with BrdU for 10, 20, or 30 min followed by a 30-min chase and replicated DNA was measured as the BrdU signal intensity via immunofluorescence. In *sml1Δ* cells, the BrdU signal intensity per nucleus increased over time, as expected with ongoing fork progression (Fig. 6A). Notably, after BrdU removal, subsequent treatment with MMS for 10, 20, or 30 min did not alter the BrdU signal, indicating that MMS had no effect on already-replicated DNA in checkpoint-proficient cells. In contrast, *rad53Δ* cells exhibited reduced BrdU incorporation over time, consistent with the slower fork progression reported in checkpoint-deficient cells [12]. Additionally, these cells displayed a marked, progressive decline in BrdU signal when exposed to MMS for 10, 20, or 30 min after BrdU removal. These findings indicate that, in the absence of Rad53, MMS treatment induces the gradual degradation of previously replicated DNA.

Given that fork arrest depends on Rad51, Rad5, and Mus81, but not Mre11, we examined the impact of losing each of these factors on nascent DNA degradation. Exo1 was also included in the analysis due to its well-known role as a suppressor of the MMS sensitivity of *rad53Δ* cells [21]. BrdU signal intensity per nucleus was quantified in cells incubated with BrdU for 30 min, followed by a 30-min chase with or without MMS. A significant decrease in BrdU signal was observed in *rad53Δ* and *rad53Δ mre11Δ* cells, whereas no such reduction occurred in *rad53Δ rad51Δ, rad53Δ mus81Δ*, *rad53Δ rad5Δ*, or *rad53Δ exo1Δ* (Fig. 6B). These results indicate that the progressive degradation of previously replicated DNA requires Rad51, Mus81, Rad5, and Exo1, but not Mre11. Thus, aberrant fork processing must be actively suppressed in WT cells to prevent the irreversible degradation of nascent DNA.

### Effect of heterologous expression of human PrimPol in yeast

In human cells, an alternative to fork reversal involves repriming of the leading strand by PrimPol, which is conserved across most eukaryotes but absent in *S. cerevisiae* (reviewed in [28, 72]). This relies on the interaction of PrimPol with the RPA complex, which accumulates on chromatin behind the advancing fork likely due to the uncoupling between leading and lagging strand synthesis [73, 74]. Thus, we wondered whether human PrimPol (*Hs*PrimPol) and yeast RPA could potentially interact. Structural models of the N-terminal domain of human RPA70 (*Hs*RPA70N) and *S. cerevisiae* RFA1 (*Sc*RFA1) support this hypothesis (Supplementary Fig. S4A). Two motifs within the RPA-binding domain of PrimPol were previously reported to directly bind to *Hs*RPA70N [73]. Superposition of *Hs*RPA70N and *Sc*RFA1 reveal a high structural similarity and indeed, equivalent residues to those that mediate the interaction with *Hs*PrimPol in *Hs*RPA70N can be found in *Sc*RFA1 (R35, K58, R93, and K95) (Supplementary Fig. S4B). Despite that T34 and S54 residues from *Hs*RPA70N, proposed to contribute to the interaction with *Hs*PrimPol [73], are poorly conserved in *Sc*RFA1, two out of the three residues in *Sc*RFA1 (K45 and K58) responsible for the interaction with yeast Dna2 and Dcd2 [75] are also located in this region and are conserved in *Hs*RPA70N (Supplementary Fig. S4B). These results support that this region of yeast RPA can mediate the interaction with other proteins, such as PrimPol, making the latter potentially functional in *S. cerevisiae*.

**Figure 7.**
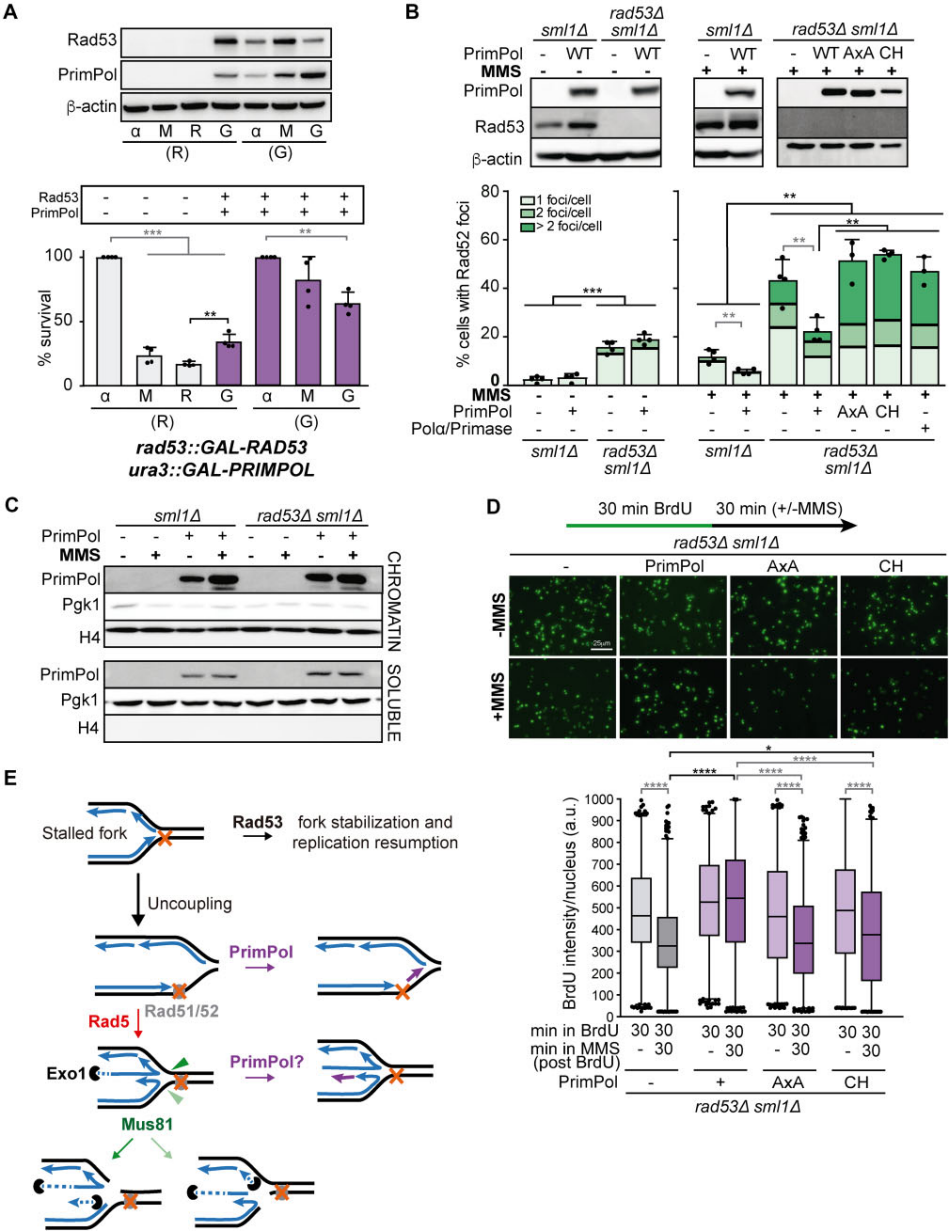
Human PrimPol expression prevents replication stress-induced DNA damage of yeast checkpoint mutants. (**A**) Immunoblot detection (top panel) of Rad53, PrimPol and loading control β-actin and cell viability (bottom panel) during re-expression experiment performed with *rad53::GAL-RAD53 ura3::GAL-PRIMPOL* (YIN095) strain. Mean and SD from four experiments (n = 4) are shown. **P < .01, ***P < .001 (two-tailed paired *t*-test). Other details as in Fig. 1D. (**B**) Immunoblot detection (top panel) of Rad53, PrimPol, and loading control β-actin expression and analysis (bottom panel) of the percentage of S/G2 cells with 1, 2, or >2 Rad52-YFP foci in asynchronous cultures of *sml1Δ* (YBG047), *sml1Δ ura3::GAL-PRIMPOL* (YIN101), *rad53Δ sml1Δ* (YBG610), *rad53Δ sml1Δ ura3::GAL-PRIMPOL* (YIN103), *rad53Δ sml1Δ ura3::GAL-PRIMPOL-AxA* (YIN114), *rad53Δ sml1Δ ura3::GAL-PRIMPOL-CH* (YIN115), and *rad53Δ sml1Δ trp1::GAL-POL1,POL12 ura3::GAL-PRI1,PRI2* (YIN167) strains transformed with pWJ1344 in unchallenged conditions or treated with MMS 0.005% for 1 h. A minimum of 100 cells were counted per experiment. Each dot represents the percentage of cells with Rad52 foci out of the total number of cells in each experiment. Mean and SD from at least three experiments (n ≥ 3) are shown. **P < .01, ***P < .001 (two-tailed unpaired *t*-test). (**C**) Recruitment of PrimPol to chromatin determined by chromatin fractionation and immunoblotting from asynchronous cultures treated or not with MMS 0.05% for 1 h. Strains used were *sml1Δ* (YBG047), *sml1Δ ura3::GAL-PRIMPOL* (YIN101), *rad53Δ sml1Δ*(YBG610), and *rad53Δ sml1Δ ura3::GAL-PRIMPOL* (YIN103). Chromatin-bound and soluble fractions are shown. Pgk1 and histone H4 were used as controls for soluble and chromatin-bound fractions, respectively. (**D**) Immunodetection of the intensity of BrdU incorporated in asynchronously growing cultures of *rad53Δ sml1Δ* (YIN124), *rad53Δ sml1Δ ura3::GAL-PRIMPOL* (YIN131), *rad53Δ sml1Δ ura3::GAL-PRIMPOL-AxA* (YIN148), and *rad53Δ sml1Δ ura3::GAL-PRIMPOL-CH* (YIN149) strains. Representative images of the different conditions are shown. Scale bar, 25 μm. A total of 612 cells per condition from two independent experiments were plotted (n = 306). *P < .05, ****P < .0001 (two-tailed Mann–Whitney U test). Other details as in Fig. 6B. (**E**) A model to explain the aberrant processing of forks that leads to irreversible fork arrest in checkpoint mutants. Upon fork stalling, Rad53 drives fork stabilization and replication resumption. However, in checkpoint-deficient conditions, Rad51 and Rad52 remain bound to the damaged DNA, potentially hindering proper DNA repair and initiating Rad5-mediated fork reversal upon extensive fork uncoupling. This fork reversal generates a substrate for Mus81 cleavage, further leading to nascent DNA degradation, a process that can be mediated by Exo1. The expression of human PrimPol in yeast can rescue irreversible fork arrest by repriming the leading strand, or by converting ssDNA into double-stranded DNA (dsDNA) at stalled, uncoupled, or reversed forks.

To investigate whether PrimPol could rescue stalled or irreversibly arrested forks in yeast, we performed re-expression experiments with a strain in which the human *PRIMPOL* complementary DNA was expressed from the *GAL* promoter. As shown in Fig. 7A, the percentage of survival after MMS treatment significantly increased twofold when both Rad53 and PrimPol were expressed after MMS removal. The viability in the checkpoint-proficient control conditions (galactose medium) was similar to that obtained in the absence of PrimPol (Fig. 1A) suggesting that human PrimPol expression in yeast does not induce MMS sensitivity. Similarly, PrimPol expression did not affect *sml1Δ* cells but slightly improved survival of *rad53Δ sml1Δ* cells under chronic MMS exposure (Supplementary Fig. S5A). We next examined the impact of PrimPol expression on Rad52 foci levels, using these as a marker for stalled or irreversibly arrested forks (Fig. 7B). Under unchallenged conditions, PrimPol expression did not alter the percentage of cells with Rad52 foci in either *sml1Δ* or *rad53Δ sml1Δ* cells. However, it significantly reduced the accumulation of Rad52 foci induced by MMS in both strains. To test if the primase activity of yeast Polα/Primase, which reprimes at the lagging strand in each Okazaki fragment, could have a similar effect when overexpressed, we built *sml1Δ* and *rad53Δ sml1Δ* strains in which all four yeast Polα/Primase complex subunits were under control of the *GAL* promoter (*trp1::GAL-POL1,POL12, ura3::GAL-PRI1,PRI2*). Overexpression of Polα/Primase was not toxic by itself but rendered *sml1Δ* cells slightly sensitive to MMS and was unable to suppress the MMS-induced lethality or the high levels of MMS-induced Rad52 foci of *rad53Δ* cells (Fig. 7B and Supplementary Fig. S5B). These results suggest that only repriming at the leading strand can rescue fork arrest.

We thus wondered whether human PrimPol was enriched on chromatin, where it would perform its repriming activity. Chromatin fractionation experiments revealed that PrimPol was enriched on chromatin independently of Rad53 (Fig. 7C and Supplementary Fig. S6A). MMS specifically increased the levels of chromatin-bound PrimPol. This agrees with PrimPol activity on chromatin being enhanced upon DNA damage, as reported in mammalian cells [76]. Chromatin-bound RPA was also increased upon MMS treatment, and at higher levels in checkpoint-deficient cells (Supplementary Fig. S6B). This is probably a consequence of the extensive fork uncoupling that occurs between leading and lagging strand synthesis in checkpoint-deficient conditions [2]. Importantly, PrimPol expression resulted in a further increase in RPA levels at chromatin, suggesting that PrimPol activity results in more or longer ssDNA gaps. These results agree with the role of PrimPol repriming the leading strand, as described in human cells, and support the idea that the effect of PrimPol in yeast checkpoint-deficient cells could be mediated by its repriming activity.

To test such a possibility, we expressed two specific PrimPol mutants: the catalytic core domain mutant AxA, which lacks all catalytic activity [26] and the Zn finger domain CH mutant, which retains polymerase activity but is selectively affected in the primase activity [27]. AxA mutant was previously reported to retain normal ssDNA binding capacity [77], and here we confirmed by EMSA that the ssDNA binding capacity of the CH mutant was not affected (Supplementary Fig. S6C), in agreement with previous results with the whole Zn finger deletion [78]. Consistently, both AxA and CH mutants were enriched on chromatin as the WT (Supplementary Fig. S6A). However, RPA levels were not increased when AxA or CH mutants were expressed (Supplementary Fig. S6B). Notably, the expression of PrimPol AxA and CH mutants had no effect on the MMS-induced Rad52 foci accumulation or in the survival of *rad53Δ sml1Δ* cells to chronic MMS exposure (Fig. 7B and Supplementary Fig. S6D). Thus, the suppressor effect of PrimPol in the MMS sensitivity of checkpoint-deficient yeast cells specifically relies on its primase activity. Altogether, these findings indicate that the primase activity of human PrimPol can rescue stalled replication forks in yeast, mitigating aberrant fork processing in checkpoint-deficient mutants.

We hypothesized that PrimPol expression might improve the survival of checkpoint mutants following MMS treatment by protecting nascent DNA from degradation. As shown in Fig. 7D, whereas *rad53Δ sml1Δ* cells expressing PrimPol AxA or CH mutants still exhibited a significant reduction in BrdU signal upon MMS treatment, WT PrimPol expression suppressed the nascent DNA degradation.

Therefore, human PrimPol can overcome replication fork arrest in yeast experiencing replication stress, through the protection of forks from nascent DNA degradation by a mechanism dependent on its primase activity.

## Discussion

In this study, we uncover that checkpoint mutants of *S. cerevisiae* do not increase the incidence of DNA damage but instead lead to the persistence of improperly processed forks. Checkpoint deficiency results in the aberrant processing of forks stalled at DNA damage leading to cell lethality. This process requires the recombination factors Rad51/52, the Rad5 ATPase and HIRAN domains, and the Mus81 nuclease, supporting a mechanism that involves fork reversal followed by cleavage that ultimately leads to nascent DNA degradation (Fig. 7E). These findings underscore two possible roles of S-phase checkpoints that would be critical to ensure cell survival following DNA damage: (i) regulating nucleases that could degrade stalled replication forks and (ii) preventing fork reversal, which generates more substrates for such and/or other nucleases.

Recombination factors were initially dismissed as contributors to fork arrest because the absence of Rad52 does not suppress the MMS sensitivity of *rad53Δ* checkpoint mutants [21]. Similarly, the absence of Rad51 does not suppress this sensitivity (Fig. 1C and I). Notably, we show here that this is because of the roles of Rad51 and Rad52 in later steps such as fork progression, consistent with previous studies [40–42]. The powerful genetic strategy of re-expression experiments has allowed the identification of these and other two factors, Mus81 and Rad5, involved in aberrant fork processing regardless of their additional functions in cell survival once the damaging agent was removed. Our study reveals that irreversible replication fork arrest can be partially mitigated by the loss of Mus81, which is essential for fork resumption [62] or by the loss of Rad5, which facilitates fork progression after MMS treatment [46] (Figs 4 and 5). A similar effect was observed with Mre11, whose loss and re-expression can suppress irreversible replication fork arrest (Fig. 3). Mre11 needs to be re-expressed after MMS removal in alignment with its role in fork resumption in checkpoint-proficient cells [79]. However, overexpression was enough to supress the MMS sensitivity of checkpoint-deficient cells in this case, and not in the case or Rad51, Mus81, or Rad5. Until now, the role of these factors in fork arrest was obscured by their additional requirement for cell survival once DNA damage is repaired.

Consistent with the idea that Rad51 contributes to the toxicity of DNA-damaging agents in checkpoint-deficient cells, the loss of *Schizosaccharomyces pombe* Rhp51 (the Rad51 homologue) has been reported to enable replication completion in checkpoint mutants treated with HU. However, this alone was insufficient to restore viability [80, 81]. Additionally, *S. pombe* Rad3 (the ATR/Mec1 homologue) has been shown to regulate the association of recombination factors with arrested forks [82]. In *S. cerevisiae*, Rad51 is recruited even to unperturbed replication forks, where it facilitates DNA damage tolerance [41]. These findings suggest that the role of Rad51 at replication forks is far more complex than simply repairing DNA lesions. In fact, we have shown that, under checkpoint-deficient conditions, Rad51 exacerbates the toxicity of replication stress caused by MMS, which is caused by Mus81 and Exo1-mediated degradation.

The current literature describes distinct recombinogenic and nonrecombinogenic roles of RAD51 during replication stress in yeast and mammalian cells such as fork stabilization, reversal, and restart [83, 84]. Mammalian RAD51, in collaboration with BRCA1, BRCA2, and Fanconi anemia proteins, plays a critical role in preventing nascent DNA degradation by MRE11 [85–88]. Interestingly, a recent study revealed that mammalian RAD51 binds apyrimidinic (AP) sites, thus blocking the access of repair glycosylases and protecting forks from MRE11-driven degradation [89]. Also, RAD52 has been shown to protect forks by restricting fork reversal [90]. While consistent with these beneficial roles, our results reveal a contrasting role for Rad51 in the absence of Rad53. Specifically, we show that Rad51 promotes nascent DNA degradation rather than preventing it, and this process is not mediated by Mre11, but by Mus81.

We infer that nascent DNA is degraded in yeast checkpoint mutants from the fact that MMS induces the loss of BrdU signal from replicated forks in the absence of Rad53 (Fig. 6). Suppression of this phenotype by deletion of the same factors that suppress the loss of cell survival (Rad51, Rad5, or Mus81) supports that irreversible fork arrest is caused by nascent DNA degradation. Moreover, this degradation phenotype can be prevented by human PrimPol expression (Fig. 7). ssDNA and RPA accumulation at forks can be used to infer DNA degradation, as reported in WT or checkpoint deficient cells [79, 91]. In agreement, we also see RPA accumulation at chromatin after DNA damage, and this is further increased in checkpoint-deficient cells (Supplementary Fig. S6B). However, since this increase cannot be suppressed by human PrimPol expression, which does suppress the loss of cell survival, we interpret that RPA accumulates at chromatin as a consequence of the extensive uncoupling between leading and lagging strand synthesis known to occur in checkpoint-deficient cells [2].

It is likely that various DNA structures can contribute to the degradation leading to irreversible fork arrest. Our data suggest that the aberrant processing of forks is partially driven by Mus81 and likely preceded by a template-switching event, such as fork reversal. This is supported by the requirement of the catalytic activity of Mus81 and the helicase and HIRAN domains of Rad5 (Figs 4 and 5). In light of our results, we propose that, among the structures that are vulnerable to nucleases, fork reversal may promote degradation by Mus81 and increase the accessibility of DNA ends, which can be further targeted by Exo1 (Fig. 7E). This aligns well with previous 2D-gel analysis suggesting that nascent strand annealing can precede DNA-end resection by Exo1 at reversed forks [18]. Our model predicts that the loss of Rad51 (or Rad52) should have a similar effect to the loss of Exo1 or Mus81 in checkpoint-deficient cells, since these factors are involved in the same pathway of irreversible replication fork arrest. In agreement, we see that either Rad51, Exo1, or Mus81 loss counteracts the degradation of nascent DNA (Fig. 6). Similarly, 2D-gel intermediates upon HU treatment revealed similar patterns in *rad53Δ* cells when either only Exo1 or both Rad52 and Exo1 or Mus81 and Exo1 were deleted [18]. Therefore, Exo1 might counteract the processing of reversed forks in WT cells as previously proposed [18] but it is toxic in *rad53Δ* cells, where it drives nucleolytic processing of intermediates [17]. This is in agreement with *exo1Δ* being identified as a suppressor of the MMS-sensitivity of *rad53Δ* cells [21]. In line with our model, fork reversal was primarily observed in checkpoint-deficient yeast cells, suggesting that it is too transient to occur under checkpoint-proficient conditions [16]. Furthermore, Mus81 is known to process various replication-associated intermediates [92], and, consistent with its potential to be harmful at forks, Mus81 activity is tightly regulated throughout the cell cycle and by the checkpoint [29, 93–95]. Mus81 has also been shown to target stalled forks leading to DSBs in checkpoint mutants of *S. pombe* [96]. Therefore, in checkpoint-deficient cells, a noncanonical or residual activity of Mus81 plus extensive fork reversal would allow Mus81 and Exo1-driven aberrant processing that contributes to the degradation phenotype reported here (Fig. 6).

Our model also aligns well with certain observations in mammalian cells. Thus, inhibition of human ATR or CHK1 was also shown to lead to DNA degradation at ongoing forks upon HU-induced replicative stress as evidenced by the loss of BrdU signal and the exposure of ssDNA at forks [97]. However, this DNA degradation was independent on fork reversal and suppressed by the loss of PrimPol [97]. It is possible that the phenomenon is different from treating with MMS in yeast cells. In contrast to our findings indicating that degradation of nascent DNA in yeast checkpoint mutants depends on Mus81 and Exo1 but not on Mre11 (Fig. 6), the degradation observed upon ATR inhibition was dependent on MRE11 and EXO1 and not on MUS81 [97]. The differential role of Mre11 at forks in checkpoint-deficient yeast and human cells may have to do with differences between the human MRN and yeast MRX complexes, particularly concerning the Nbs1 and Xrs2 subunits which are less conserved than Mre11 and Rad50 across species [98]. Alternatively, the differences found could also depend on the DNA damage source and extent as well as the genetic background. Indeed, MRE11-independent DNA degradation and ssDNA accumulation behind forks, relying on EXO1, has been reported in checkpoint-proficient human cells [99].

Moreover, in line with our observations in yeast, human MUS81 was reported to generate DSBs at replication forks not only in checkpoint-proficient cells upon prolonged replication stress treatments but also in checkpoint-deficient cells upon acute treatments [100]. MUS81 has been implicated in DNA degradation under certain conditions also in human cells, such as upon depletion of SMARCAL1 or BRCA1/2 or SIN3 [101–103] and multiple reports suggest that DNA degradation at forks requires fork reversal, which in humans is driven by the Rad5 orthologue HLTF, or by other SNF2-family chromatin remodelers like SMARCAL1 or ZRANB3 [104, 105]. It is described as a dynamic process that appears to precede fork collapse but also plays a critical role in fork protection [106, 107]. In line with this, we found that the HIRAN domain of Rad5 not only contributes to irreversible fork arrest but also plays a key role in fork resumption following MMS removal (Fig. 5). Similarly, the HIRAN domain of HLTF is critical for fork reversal and regulating fork progression in human cells [108]. The observed requirement of Rad51 for fork arrest could therefore be a consequence of its role in facilitating fork reversal [109]. In summary, fork reversal likely serves to protect forks in WT cells, while it creates a substrate for Mus81 cleavage in checkpoint-deficient cells, ultimately leading to irreversible replication fork arrest.

Our results indicate that Mph1, Mte1, Pol32, Rad54, Sae2, Sgs1, Shu1, and Srs2 have no role in the MMS sensitivity of *rad53Δ* cells. The lack of suppression by Pol32 rules out the process of break-induced replication in fork arrest upon MMS. Yet, the specific role for these and other factors subjected to other sources of DNA damage might be different. For instance, the role of Mph1 at stalled forks, which, like its human ortholog FANCM, can catalyse fork reversal *in vitro* [110, 111], is different for interstrand crosslinks versus MMS [112]. Moreover, the suppressor effect of *exo1Δ* and other deletions, such as Rpd3L, on the MMS-induced sensitivity of *rad53Δ* cells, was not observed at the same level upon HU [21, 23] and thus, different processing factors might act upon different sources of DNA damage. Additionally, it is worth considering that not all fork processing necessarily leads to lethality. For instance, whereas Sae2 was reported to process replication intermediates upon HU [18], we see no suppression of the MMS sensitivity of *rad53Δ* cells upon its loss. In contrast, whereas we observed that Mus81 loss during MMS treatment and subsequent re-expression rescues the viability of checkpoint-deficient cells, it was reported not to affect the replication intermediates in 2D-gel experiments upon HU [18]. Our re-expression strategy to test survival serves to clarify which processing activities mediate the toxicity of MMS in checkpoint-deficient cells.

Our re-expression experiments also revealed that overexpression of *MRE11* or *RAD50* counteract fork arrest in a nuclease-independent manner (Fig. 3 and Supplementary Fig. S3). One possibility is that *MRE11* or *RAD50* overexpression could promote the recruitment of other factors that are relevant for fork protection or accurate processing. Along this line, the MRN complex, but not the nuclease activity of Mre11, has been shown to assist in fork stabilization in *S. pombe* by facilitating Rad52 recruitment [82]. Alternatively, or in addition, *MRE11* or *RAD50* overexpression could protect the DNA ends of reversed forks or fork stability. In line with this, MRX, but not other DNA-end processing factors such as Sae2, has been reported to be recruited to stalled forks to promote the stability of the replisome [113]. Interestingly, and similar to our results (Fig. 3 and Supplementary Fig. S3), this was shown for Mre11 independent on its nuclease activity, and for Rad50, but was less clear in the case of Xrs2 [113]. It is worth noticing that MRX supports the stability of the replisome by the cohesion between sister chromatids [113, 114] and that the Rpd3L histone deacetylase complex, which influences the repair of broken forks by promoting sister chromatid cohesion [115], was identified among the stronger suppressors of the MMS-sensitivity of *rad53Δ* cells [23]. Thus, it is tempting to speculate that the suppression we see by *MRE11* or *RAD50* overexpression may be related with a function in sister chromatid cohesion.

Remarkably, we also show that when expressed in yeast, human PrimPol can counteract nascent DNA degradation and partially rescue arrested forks, even after their aberrant processing, leading to ssDNA accumulation as revealed by increased RPA on chromatin (Fig. 7 and Supplementary Figs S4 and S6). This suggests that checkpoint-induced upregulation of the repriming activity of PrimPol could serve as a natural strategy to prevent or counteract the degradation of reversed forks in human cells, as has been observed in BRCA-deficient cells following acute chemotherapeutic treatment [116]. PrimPol is phosphorylated by CHK1 upon different sources of replication stress to promote repriming at the expense of ssDNA gap formation [117]. While PrimPol repriming activity is critical for resolving various types of DNA lesions, it is absent in certain organisms, such as *S. cerevisiae* and *Caenorhabditis elegans* [26, 27, 116, 118–122]. Our findings not only open the door to studying human PrimPol mutations using yeast as an *in vivo* test-tube but also provide valuable insights into the possible intermediates that arise in checkpoint mutants when encountering DNA lesions.

The effect of PrimPol in yeast fork arrest could be related with the protection of accessible DNA ends from nucleases. However, the dependency on the primase activity suggests an active PrimPol role being required for the suppression. The ability of PrimPol to synthesize DNA from any ssDNA template also raises the possibility that its effects results from the conversion into dsDNA at stalled or reversed forks or even from filling ssDNA gaps at the leading or lagging strand behind arrested forks. However, the increased chromatin-bound RPA upon PrimPol expression (Supplementary Fig. S6B) supports that it directly reprimes the leading strand ahead of stalled forks in yeast, as in human cells. Indeed, we show that overexpression of the yeast Polα/Primase complex, which is known to reprime the lagging strand, cannot promote MMS survival (Fig. 7 and Supplementary Fig. S5). This agrees with structure and *in vitro* experiments indicating that yeast Polα/Primase complex is unable to reprime the leading strand [123]. PrimPol rescues arrested forks by interacting with RPA, which accumulates upon uncoupling between leading and lagging strand synthesis [73, 74]. In *rad53Δ* cells, the extensive uncoupling of leading and lagging strand synthesis (see [2] and Supplementary Fig. S6B) would facilitate PrimPol repriming, thus preventing further processing that leads to DNA degradation. Thus, irreversible replication fork arrest likely begins with the uncoupling of leading and lagging strand synthesis, a process that occurs in checkpoint-deficient cells (Fig. 7E).

The key question remains: what specific proteins or activities does the checkpoint target to promote fork stability? In line with current literature, we propose that the checkpoint's essential function involves the regulation of multiple factors. This view is consistent with proteomic data in human cells that show that ATR directly regulates the activity of multiple fork remodeling enzymes to prevent fork collapse [124]. Of note, this regulation does not involve changes in gene expression since *de novo* protein synthesis was reported not to be required for HU or MMS survival [14, 23]. We report here that Exo1 and Mus81 participate in nascent DNA degradation (Fig. 6), in agreement with previous reports [16–20]. Thus, we believe that checkpoint regulation of these and possibly other nuclease activities may contribute to their action at forks. Checkpoint activation by HU or MMS is known to slow down DSB end resection [40, 125], a process driven by Exo1, Dna2, Sae2, and Sgs1, all of which are regulated by the checkpoint (reviewed in [126]). Mus81 is also indirectly regulated by the checkpoint [127]. Moreover, the Rad53 fission yeast counterpart Cds1 phosphorylates Mus81 to prevent the accumulation of toxic structures [128, 129] and Dna2 to counteract fork reversal [130]. Thus, checkpoint-mediated regulation of nucleases involved in DNA end resection or other steps in homologous recombination could contribute to prevent irreversible fork arrest in budding yeast. Additionally, by restricting fork uncoupling or reversal, the checkpoint helps prevent the formation of substrates for DNA degradation.

Altogether, our results support the model in which the essential function of the checkpoint is to prevent the aberrant degradation of nascent DNA upon encountering DNA lesions caused by endogenous or exogenous DNA damage.

## Supplementary Material

gkaf707_Supplemental_File

## Data Availability

The data underlying this article will be shared on reasonable request to the corresponding author.
