# Supplementary material for: S-phase checkpoint protects from aberrant replication fork processing and degradation
Source: Nucleic Acids Res. 2025 Jul 30;53(14):gkaf707. doi: 10.1093/nar/gkaf707 (PMC12309369; doi:10.1093/nar/gkaf707)
Supplement: gkaf707_Supplemental_File [file gkaf707_supplemental_file.pdf]

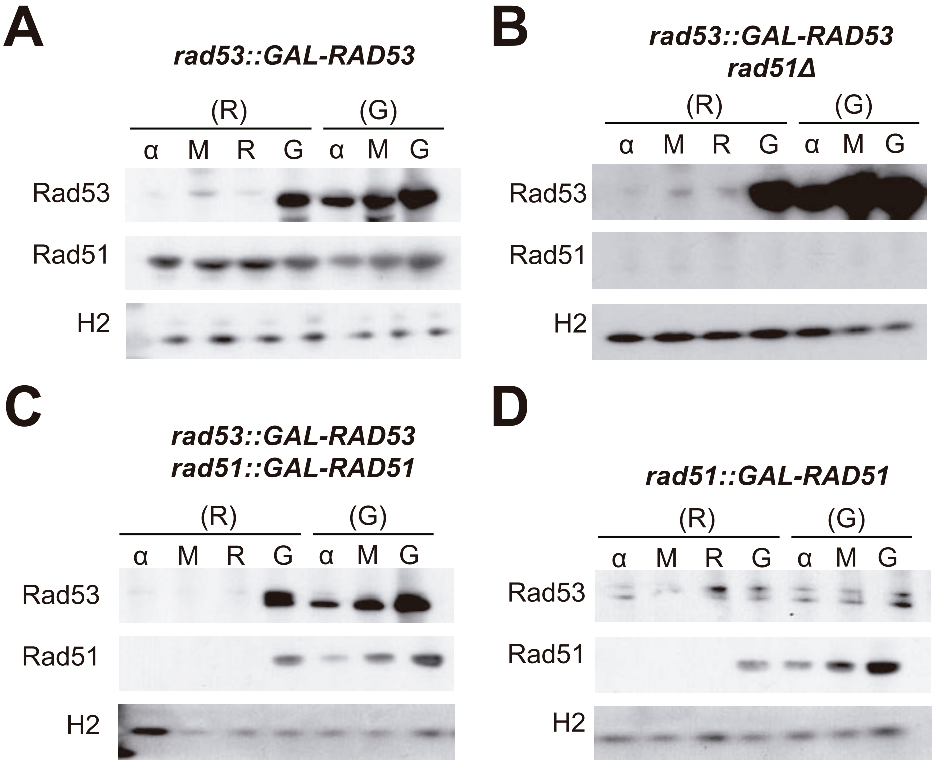

Figure S1. Rad53 and Rad51 levels during re-expression experiments.
Immunoblot detection of Rad53, Rad51 and H2 as loading control at the indicated stages of the re-expression experiments described in Figure 1D .

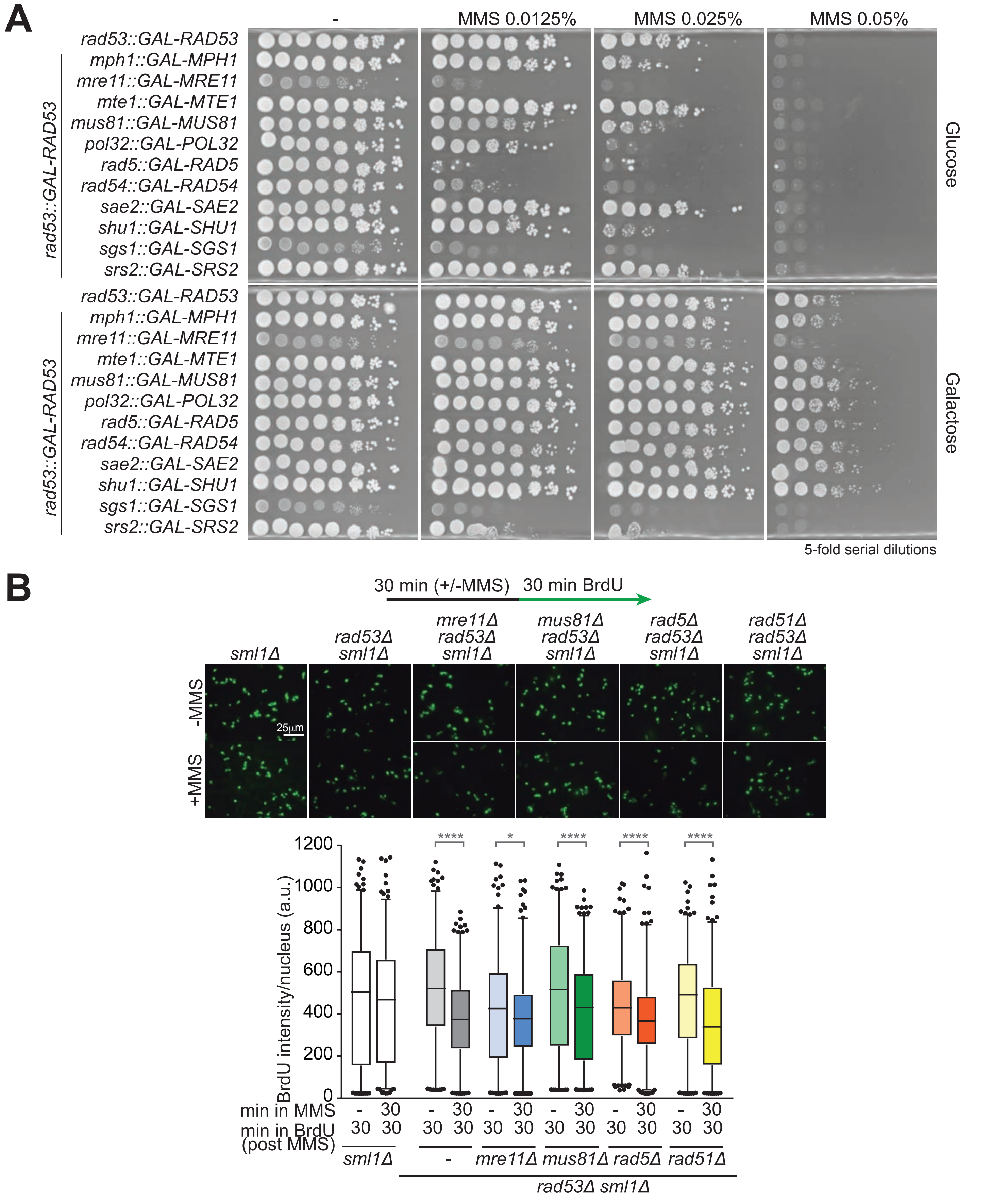

Figure S2. MMS sensitivity and defects in replication resumption upon loss of Rad53 and selected factors. **(A)** Sensitivity to chronic treatment with different concentrations of MMS of the indicated strains, assayed by 5-fold serial dilutions. **(B)** Schematic of the experimental set-up (top panel): cells were incubated with or without MMS 0.05% for 30 min, treated with 2.5% sodium thiosulfate to inactivate MMS and then pulse-labelled with 200 μM BrdU for 30 min, washed and released in fresh YPAD medium without BrdU for 30 min. Immunodetection (bottom panel) of the intensity of BrdU incorporated in asynchronously growing cultures of *sml1*Δ (YIN122), *rad53*Δ *sml1*Δ (YIN124), *rad53*Δ *sml1*Δ *mre11*Δ (YIN129), *rad53*Δ *sml1*Δ *mus81*Δ (YIN126), *rad53*Δ *sml1*Δ *rad5*Δ (YIN130) and *rad53*Δ *sml1*Δ *rad51*Δ (YIN174) strains. Representative images of the different conditions are shown. Scale bar, 25 μm. Box and whiskers (2.5-97.5 percentile) plots show the distribution of BrdU intensities per yeast nuclei. The central horizontal line represents the median value. A total of 392 cells per condition from 2 independent experiments were plotted (n = 392). *p < 0.05, ****p < 0.0001 (two-tailed Mann-Whitney U-test). Grey stars denote significant decreases.

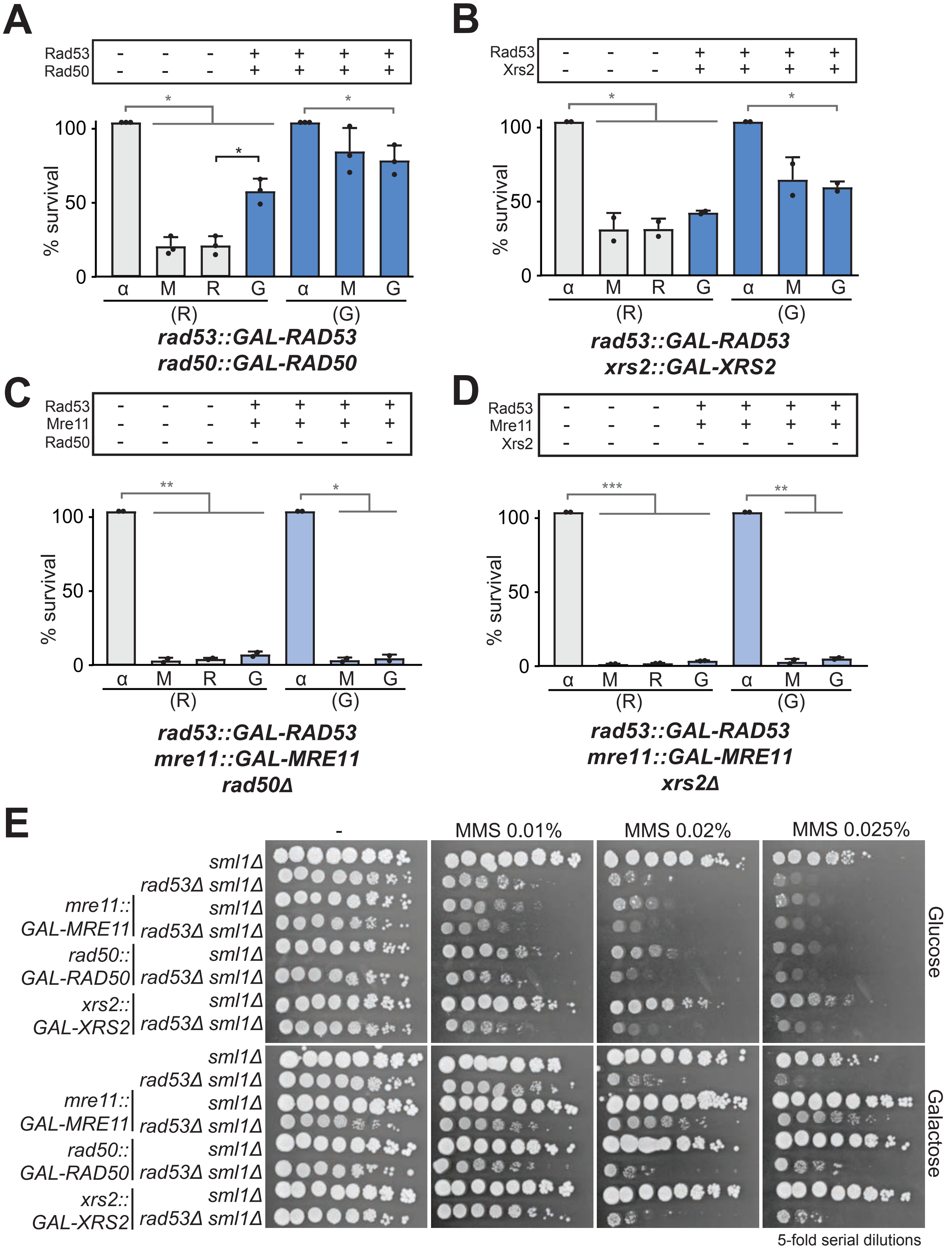

Figure S3. Effect of MRX overexpression or loss in irreversible replication fork arrest.
Cell viability measured during re-expression experiments performed with *rad53::GAL-RAD53 rad50::GAL-RAD50* (YIN158) **(A)**, *rad53::GAL-RAD53 xrs2::GAL-XRS2* (YIN157) **(B)**, *rad53::GAL-RAD53 mre11::GAL-MRE11 rad50Δ* (YIN155) **(C)** and *rad53::GAL-RAD53 mre11::GAL-MRE11 xrs2Δ* (YIN153) **(D)** strains. Other details as in Fig 1D. Each dot represents the percentage of survival for each time point per experiment. Mean and SD from at least 2 experiments ($n \geq 2$) are shown. *$p < 0.05$, **$p < 0.01$, ***$p < 0.001$ (two-tailed paired t-test). Black stars denote significant increases, whereas grey stars denote significant decreases. **(E)** Sensitivity to chronic treatment with different concentrations of MMS of *sml1Δ* (YBG621), *rad53Δ sml1Δ* (YBG501), *sml1Δ mre11::GAL-MRE11* (YIN053), *rad53Δ sml1Δ mre11::GAL-MRE11* (YIN047), *sml1Δ rad50::GAL-RAD50* (YIN170), *rad53Δ sml1Δ rad50::GAL-RAD50* (YIN171), *sml1Δ xrs2::GAL-XRS2* (YIN172) and *rad53Δ sml1Δ xrs2::GAL-XRS2* (YIN173) strains, assayed by 5-fold serial dilutions.

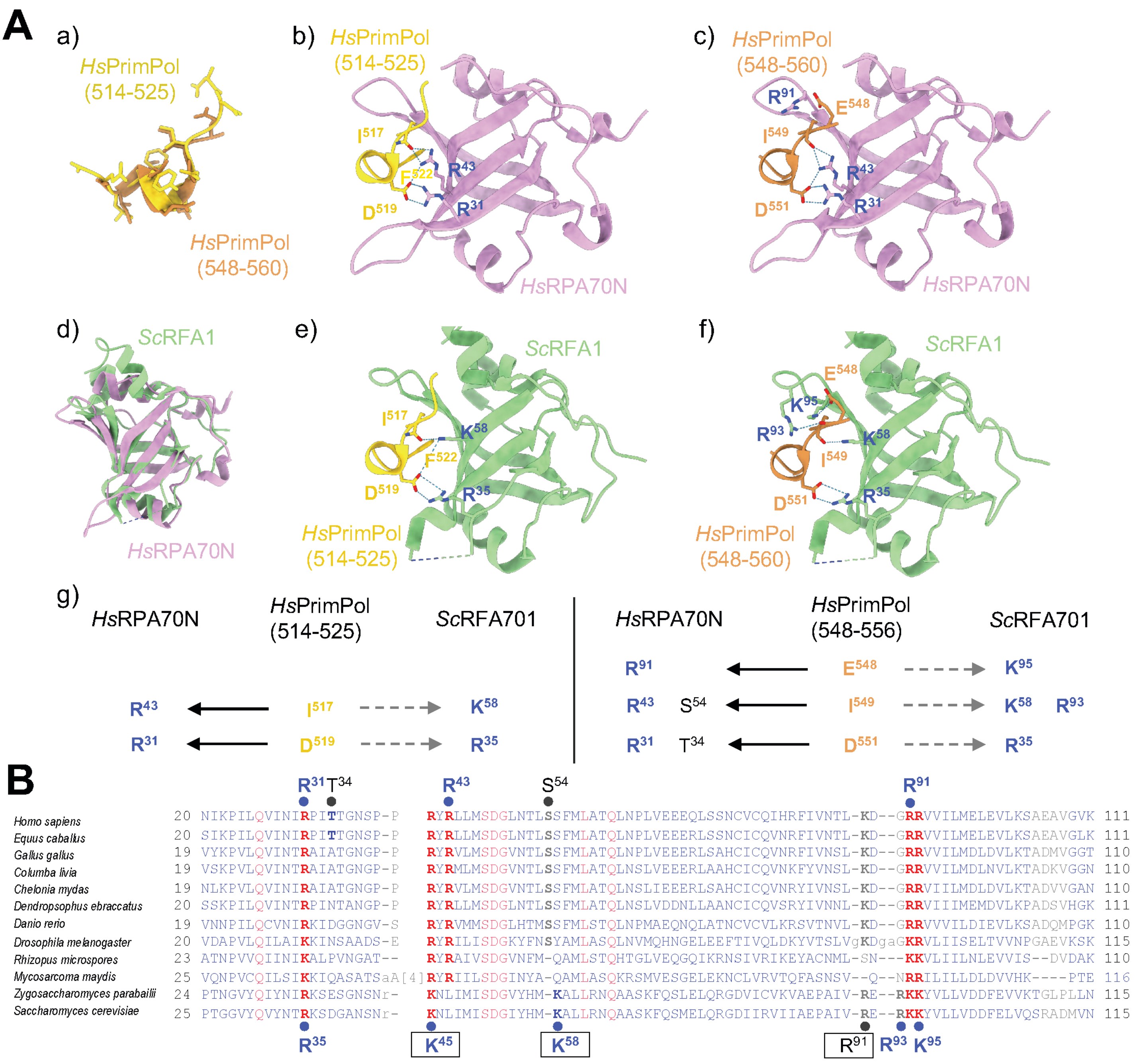

Figure S4. Human PrimPol potentially interacts with yeast RFA1 based in molecular modelling analysis and multiple sequence alignments.
(A) Structural models illustrating the proposed interaction between human PrimPol (*Hs*PrimPol) and *Saccharomyces cerevisiae* RFA1 (*Sc*RFA1): a) Superposition of *Hs*PrimPol residues 514-525 (yellow, PDB: 5n85) and 548-556 (brown, PDB: 5n8a), both involved in binding the N-terminal domain of *Hs*RPA70 (*Hs*RPA70N), emphasizing their structural similarity; b) Experimental structure outlining the interaction between *Hs*PrimPol residues 514-525 and two specific arginines (blue) of *Hs*RPA70N (PDB: 5n85); c) Experimental structure outlining the interaction between *Hs*PrimPol residues 548-556 and three specific arginines (blue) of the *Hs*RPA70N (PDB: 5n8a); d) Structural superposition of *Hs*RPA70N (from PDB: 5n85) and *Sc*RFA1 (from PDB: 8b4j), emphasizing their structural similarity; e) Structural model of the potential interaction between *Hs*PrimPol residues 514-525 (from PDB: 5n85) and R35 and K58 of *Sc*RFA1 (from PDB: 8b4j); f) Structural model of the potential interaction between *Hs*PrimPol residues 548-556 (from PDB: 5n8a) and R35, K58, R93 and K95 of *Sc*RFA1 (from PDB: 8b4j); g) Summary of the experimental interactions (Guilliam et al., 2017; indicated with black arrows) between *Hs*PrimPol and *Hs*RPA70, and the potential equivalent interactions with *Sc*RFA1 (grey/dashed arrows). **(B)** Partial sequence alignment of RPA-like proteins, in the region that *Hs*PrimPol interacts. Highly conserved amino acids are shown in red while moderately conserved are shown in blue. Experimental and proposed residues involved in the interaction with *Hs*PrimPol are highlighted in bold. Residues T34 and S54 from *Hs*RPA70, proposed to be involved in the interaction with *Hs*PrimPol are shown in black. Residues K45, K58 and R91 from *Sc*RFA1, previously reported to be responsible for the interactions with Dna2 and Ddc2, are shown in boxes.

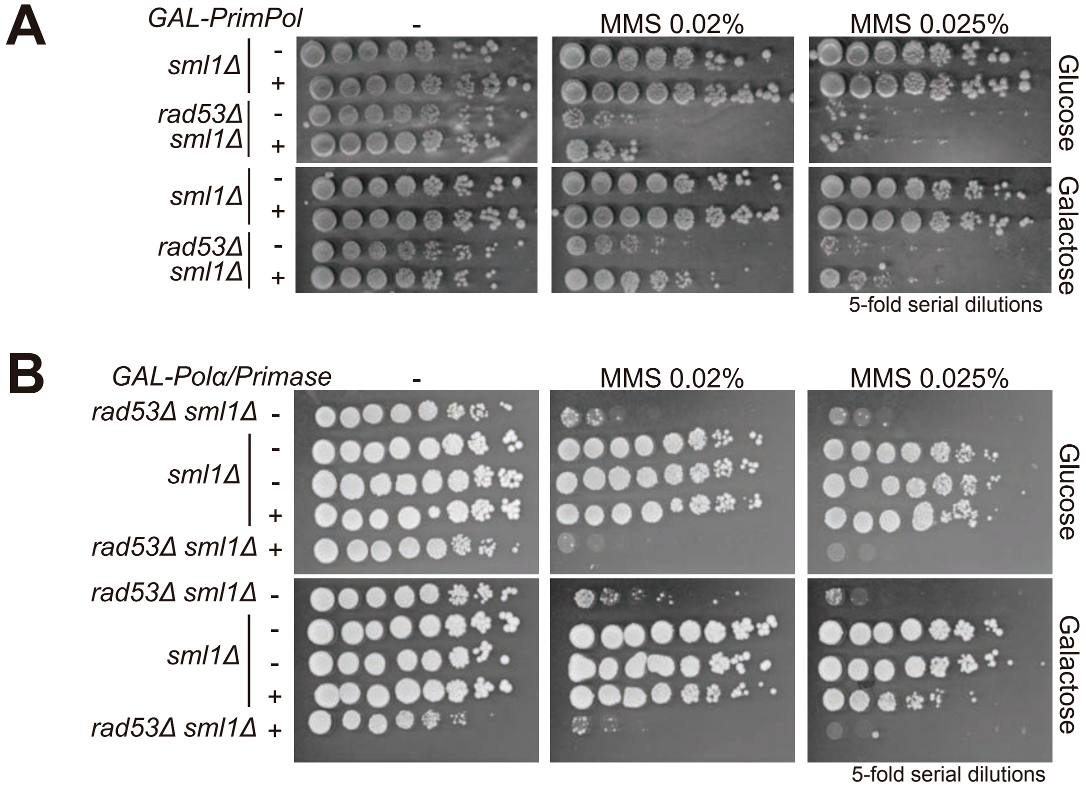

Figure S5. Effect of PrimPol expression or Polα/Primase overexpression on yeast MMS sensitivity. **(A)** Sensitivity to chronic treatment with different concentrations of MMS of *sml1*Δ (YBG047), *sml1*Δ *ura3::GAL-PRIMPOL* (YIN101), *rad53*Δ *sml1*Δ (YBG610) and *rad53*Δ *sml1*Δ *ura3::GAL-PRIMPOL* (YIN103) strains, assayed by 5-fold serial dilutions. **(B)** Sensitivity to chronic treatment with different concentrations of MMS of *rad53*Δ *sml1*Δ (YBG610), *sml1*Δ (YBG047), *sml1*Δ *trp1::GAL-POL1,POL12 ura3::GAL-PRI1,PRI2* (YIN166) and *rad53*Δ *sml1*Δ *trp1::GAL-POL1,POL12 ura3::GAL-PRI1,PRI2* (YIN167) strains, assayed by 5-fold serial dilutions.

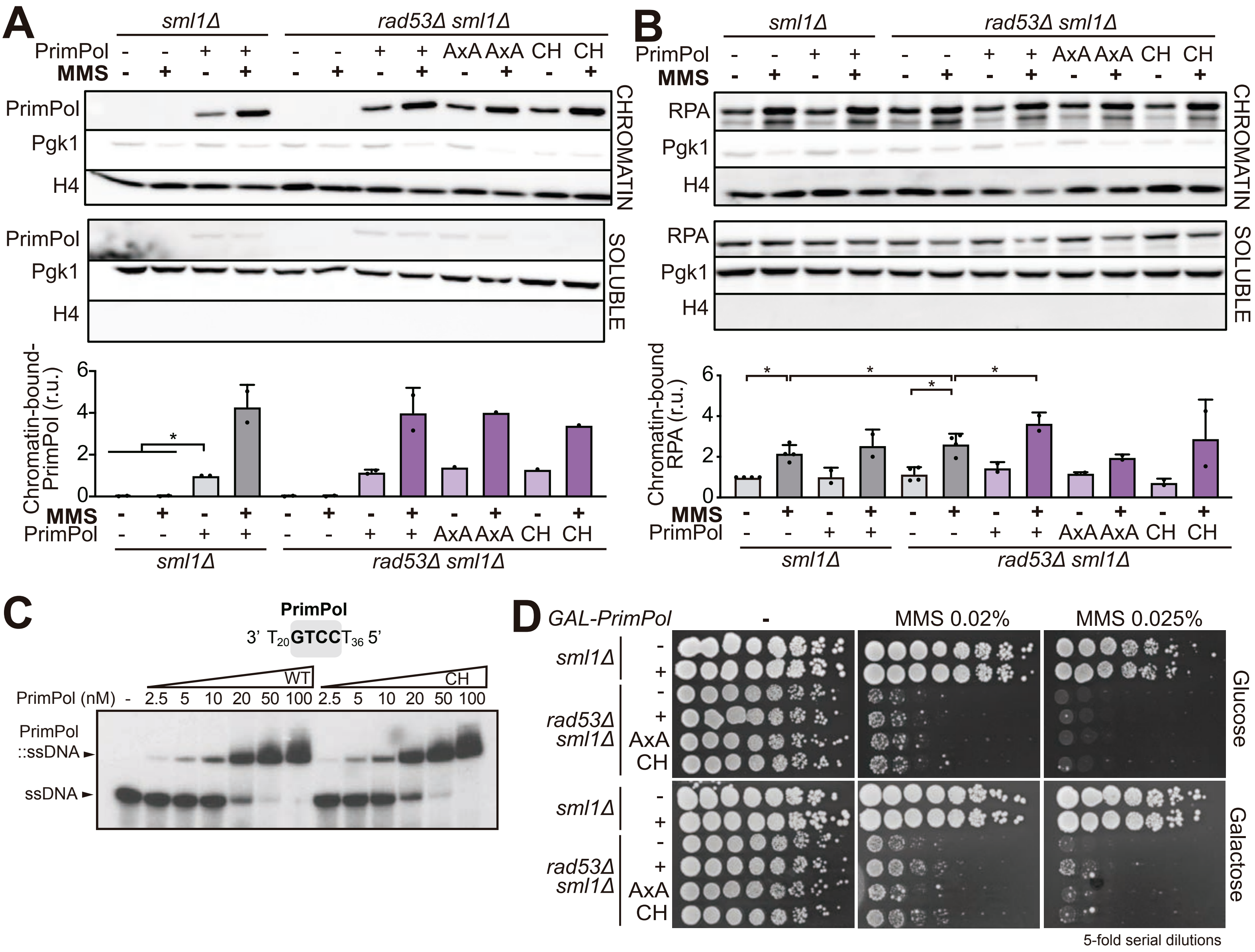

Figure S6. PrimPol and RPA recruitment to chromatin and effect on MMS sensitivity upon the expression of wild-type or PrimPol catalytic mutants in yeast.

Recruitment of PrimPol **(A)** and RPA **(B)** to chromatin (top panel) determined by chromatin fractionation and immunoblotting from asynchronous cultures treated or not with MMS 0.05% for 1 hour. Strains used were *sml1*Δ (YBG047), *sml1*Δ *ura3::GAL-PRIMPOL* (YIN101), *rad53*Δ *sml1*Δ (YBG610), *rad53*Δ *sml1*Δ *ura3::GAL-PRIMPOL* (YIN103), *rad53*Δ *sml1*Δ *ura3::GAL-PRIMPOL-AxA* (YIN114) and *rad53*Δ *sml1*Δ *ura3::GAL-PRIMPOL-CH* (YIN115). Chromatin-bound and soluble fractions are shown. Pgk1 and histone H4 were used as controls for soluble and chromatin-bound fractions, respectively. Quantification (bottom panel) of chromatin-bound PrimPol (A) and RPA (B). **(C)** EMSA showing the interaction of wild-type PrimPol (WT) or CH mutant (2.5, 5, 10, 20, 50, 100 nM) with [γ-32P]-labelled GTCC oligonucleotide (60-mer; 1 nM) in the absence of metal cofactor. **(D)** Sensitivity to chronic treatment with different concentrations of MMS of *sml1*Δ (YBG047), *sml1*Δ *ura3::GAL-PRIMPOL* (YIN101), *rad53*Δ *sml1*Δ (YBG610), *rad53*Δ *sml1*Δ *ura3::GAL-PRIMPOL* (YIN103), *rad53*Δ *sml1*Δ *ura3::-GAL-PRIMPOL-AxA* (YIN114) and *rad53*Δ *sml1*Δ *ura3::GAL-PRIMPOL-CH* (YIN115) strains, assayed by 5-fold serial dilutions.

In A and B, each dot represents the amount of protein in the chromatin fraction per experiment. Protein signals are quantified relative to histone H4 as loading control for the chromatin fraction and normalized to control samples. Mean and SD from at least 1 experiment ($n \geq 1$) are shown. Black stars denote significant increases. *$p < 0.05$ (two-tailed paired t-test).

Supplementary Table S1_ Strains used in this study

Name	Genotype	Source
YBP249	*MATa ade2-1 can1-100 his3-11,15 leu2-3,112 trp1-1 ura3-1 bar1Δ RAD5+*	(62)
YBG621	*MATa sml1Δ::URA3 RAD5+*	This study
YBG047	*MATa sml1Δ::HPH RAD5+*	This study
YBG501	*MATa sml1Δ::URA3 rad53Δ::HIS3 RAD5+*	This study
YBG610	*MATa sml1Δ::HPH rad53Δ::HIS3 RAD5+*	This study
YIN101	*MATa sml1Δ::HPH ura3::GAL-PRIMPOL RAD5+*	This study
YIN103	*MATa rad53Δ::HIS3 sml1Δ::HPH ura3::GAL-PRIMPOL RAD5+*	This study
YIN114	*MATa rad53Δ::HIS3 sml1Δ::HPH ura3::GAL-PRIMPOL-AxA RAD5+*	This study
YIN115	*MATa rad53Δ::HIS3 sml1Δ::HPH ura3::GAL-PRIMPOL-CH RAD5+*	This study
YIN001	*MATa rad53::GAL-RAD53::KAN bar1Δ RAD5+*	This study
YIN095	*MATa rad53::GAL-RAD53::KAN ura3::GAL-PRIMPOL bar1Δ RAD5+*	This study
YIN166	*MATa sml1Δ::HPH trp1::GAL-POL1,POL12 ura3::GAL-PRI1,PRI2 RAD5+*	This study
YIN167	*MATa rad53Δ::HIS3 sml1Δ::HPH trp1::GAL-POL1,POL12 ura3::GAL-PRI1,PRI2 RAD5+*	This study
YJT72	*MATa sml1Δ::URA3 rad5-535*	(12)
YBG437	*MATa sml1Δ::URA3 rad53Δ::HIS3 rad5-535*	This study
YLD142	*MATa sml1Δ::URA3 rad51Δ::HIS3 rad5-535*	This study
YBG486	*MATa sml1Δ::URA3 rad51Δ::HIS3 rad53Δ::KAN rad5-535*	This study
YBG572	*MATa sml1Δ::URA3 ARS305Δ::KAN ARS608Δ::HIS3 ARS609Δ::TRP1 rad53::GAL-RAD53::LEU2 rad51Δ::NAT rad5-535*	This study
YBG571	*MATa sml1Δ::URA3 ARS305Δ::KAN ARS608Δ::HIS3 ARS609Δ::TRP1 rad53::GAL-RAD53::LEU2 rad51::GAL-RAD51::NAT rad5-535*	This study
YBG573	*MATa sml1Δ::URA3 ARS305Δ::KAN ARS608Δ::HIS3 ARS609Δ::TRP1 rad51::GAL-RAD51::NATNT2 rad5-535*	This study
YIN138	*MATa sml1Δ::URA3 rad51::GAL-RAD51::NAT RAD5+*	This study
YIN139	*MATa rad53Δ::HIS3 sml1Δ::URA3 rad51::GAL-RAD51::NAT RAD5+*	This study
YIN006	*MATa exo1::GAL-EXO1::NAT bar1Δ RAD5+*	This study
YIN010	*MATa rad53::GAL-RAD53::KAN exo1::GAL-EXO1::NAT bar1Δ RAD5+*	This study
YIN003	*MATa mus81::GAL-MUS81::NAT bar1Δ RAD5+*	This study
YIN005	*MATa rad53::GAL-RAD53::KAN mus81::GAL-MUS81::NAT bar1Δ RAD5+*	This study
YIN020	*MATa mre11::GAL-MRE11::NAT bar1Δ RAD5+*	This study
YIN021	*MATa rad53::GAL-RAD53::KAN mre11::GAL-MRE11::NAT bar1Δ RAD5+*	This study
YIN141	*MATa rad5::GAL-RAD5::NAT bar1Δ*	This study
YIN112	*MATa rad53::GAL-RAD53::KAN rad5::GAL-RAD5::NAT bar1Δ*	This study
YIN049	*MATa sae2::GAL-SAE2::NAT bar1Δ RAD5+*	This study
YIN056	*MATa rad53::GAL-RAD53::KAN sae2::GAL-SAE2::NAT bar1Δ RAD5+*	This study
YIN008	*MATa sgs1::GAL-SGS1::NAT bar1Δ RAD5+*	This study
YIN011	*MATa rad53::GAL-RAD53::KAN sgs1::GAL-SGS1::NAT bar1Δ RAD5+*	This study
YIN023	*MATa pol32::GAL-POL32::NAT bar1Δ RAD5+*	This study
YIN025	*MATa rad53::GAL-RAD53::KAN pol32::GAL-POL32::NAT bar1Δ RAD5+*	This study
YIN035	*MATa srs2::GAL-SRS2::NAT bar1Δ RAD5+*	This study
YIN062	*MATa rad53::GAL-RAD53::KAN srs2::GAL-SRS2::NAT bar1Δ RAD5+*	This study
YIN016	*MATa mph1::GAL-MPH1::NAT bar1Δ RAD5+*	This study
YIN018	*MATa rad53::GAL-RAD53::KAN mph1::GAL-MPH1::NAT bar1Δ RAD5+*	This study
YIN026	*MATa rad54::GAL-RAD54::NAT bar1Δ RAD5+*	This study
YIN028	*MATa rad53::GAL-RAD53::KAN rad54::GAL-RAD54::NAT bar1Δ RAD5+*	This study
YIN043	*MATa mte1::GAL-MTE1::NAT bar1Δ RAD5+*	This study
YIN060	*MATa rad53::GAL-RAD53::KAN mte1::GAL-MTE1::NAT bar1Δ RAD5+*	This study
YIN039	*MATa shu1::GAL-SHU1::NAT bar1Δ RAD5+*	This study
YIN058	*MATa rad53::GAL-RAD53::KAN shu1::GAL-SHU1::NAT bar1Δ RAD5+*	This study
YIN077	*MATa rad53::GAL-RAD53::KAN mre11Δ::NAT bar1Δ RAD5+*	This study
YIN047	*MATa rad53Δ::HIS3 sml1Δ::URA3 mre11::GAL-MRE11::NAT RAD5+*	This study
YIN081	*MATa rad53::GAL-RAD53::KAN mre11::GAL-mre11-H125N::NAT RAD5+*	This study

YIN140	*MATa rad53::GAL-RAD53::KAN mre11-H125N bar1Δ RAD5+*	This study
YIN053	*MATα sml1Δ::URA3 mre11::GAL-MRE11::NAT RAD5+*	This study
YIN109	*MATα sml1Δ::URA3 mre11::GAL-mre11-H125N::NAT RAD5+*	This study
YIN111	*MATa rad53Δ::HIS3 sml1Δ::URA3 mre11::GAL-mre11-H125N::NAT RAD5+*	This study
YIN155	*MATa rad53::GAL-RAD53::KAN mre11::GAL-MRE11::NAT rad50Δ::HYG bar1Δ RAD5+*	This study
YIN153	*MATa rad53::GAL-RAD53::KAN mre11::GAL-MRE11::NAT xrs2Δ::HYG bar1Δ RAD5+*	This study
YIN158	*MATa rad53::GAL-RAD53::KAN rad50::GAL-RAD50::NAT bar1Δ RAD5+*	This study
YIN157	*MATa rad53::GAL-RAD53::KAN xrs2::GAL-XRS2::NAT bar1Δ RAD5+*	This study
YIN170	*MATa sml1Δ::HPH rad50::GAL-RAD50::NAT RAD5+*	This study
YIN171	*MATa rad53Δ::HIS3 sml1Δ::HPH rad50::GAL-RAD50::NAT RAD5+*	This study
YIN172	*MATa sml1Δ::HPH xrs2::GAL-XRS2::NAT RAD5+*	This study
YIN173	*MATa rad53Δ::HIS3 sml1Δ::HPH xrs2::GAL-XRS2::NAT RAD5+*	This study
YIN074	*MATa rad53::GAL-RAD53::KAN mus81Δ::NAT RAD5+*	This study
YIN030	*MATa rad53Δ::HIS3 sml1Δ::URA3 mus81::GAL-MUS81::NAT RAD5+*	This study
YIN083	*MATa rad53::GAL-RAD53::KAN mus81-dd bar1Δ RAD5+*	This study
YIN085	*MATa rad53::GAL-RAD53::KAN ura3::GAL1,10-MUS81 bar1Δ RAD5+*	This study
YIN088	*MATa rad53::GAL-RAD53::KAN mus81-dd ura3::GAL1,10-MUS81 bar1Δ RAD5+*	This study
YIN135	*MATa sml1Δ::URA3 mus81::GAL-MUS81::NAT RAD5+*	This study
YIN142	*MATa sml1Δ::URA3 mus81-dd RAD5+*	This study
YIN143	*MATa rad53Δ::HIS3 sml1ΔURA3 mus81-dd RAD5+*	This study
YBG592	*MATa ARS305Δ::kanMX ARS608Δ::HIS3 ARS609Δ::TRP1 rad53::GAL-RAD53::LEU2 rad5Δ::HPH*	This study
YIN093	*MATa rad53::GAL-RAD53::KAN rad5-I916A bar1Δ*	This study
YIN094	*MATa rad53::GAL-RAD53::KAN rad5-QD::HYG bar1Δ*	This study
YIN080	*MAT rad53::GAL-RAD53::KAN rad5-HIRAN bar1Δ*	This study
YIN089	*MATa rad53::GAL-RAD53::KAN ura3::GAL1,10-RAD5 bar1Δ*	This study
YIN091	*MATa rad53::GAL-RAD53::KAN rad5-HIRAN ura3::GAL1,10-RAD5 bar1Δ*	This study
YIN136	*MATa sml1Δ::URA3 rad5::GAL-RAD5::NAT*	This study
YIN137	*MATa rad53Δ::HIS3 sml1Δ::URA3 rad5::GAL-RAD5::NAT*	This study
YIN104	*MATa sml1Δ::URA3 rad5-HIRAN*	This study
YIN105	*MATa rad53Δ::HIS3 sml1Δ::URA3 rad5-HIRAN*	This study
YIN146	*MATa sml1Δ::URA3 rad5-QD::HYG*	This study
YIN147	*MATa rad53Δ::HIS3 sml1Δ::URA3 rad5-QD::HYG*	This study
YIN107	*MATa sml1Δ::URA3 rad5-I916A*	This study
YIN108	*MATa rad53Δ::HIS3 sml1Δ::URA3 rad5-I916A*	This study
BT1	*MATa bar1Δ::LEU2 ura3-1::URA3-GPD-hsvTKCO-ADH-hENT1CO(5x) RAD5+*	(52)
YIN122	*MATa ura3-1::URA3-GPD-hsvTKCO-ADH-hENT1CO(5x) sml1Δ::NAT bar1Δ RAD5+*	This study
YIN124	*MATa ura3-1::URA3-GPD-hsvTKCO-ADH-hENT1CO(5x) rad53Δ::KAN sml1Δ::NAT bar1Δ RAD5+*	This study
YIN174	*MATa ura3-1::URA3-GPD-hsvTKCO-ADH-hENT1CO(5x) rad53Δ::KAN sml1Δ::NAT rad51Δ::HPH bar1Δ RAD5+*	This study
YIN129	*MATa ura3-1::URA3-GPD-hsvTKCO-ADH-hENT1CO(5x) rad53Δ::KAN sml1Δ::NAT mre11Δ::HPH bar1Δ RAD5+*	This study
YIN126	*MATa ura3-1::URA3-GPD-hsvTKCO-ADH-hENT1CO(5x) rad53Δ::KAN sml1Δ::NAT mus81Δ::HPH bar1Δ RAD5+*	This study

YIN130	*MATa ura3-1::URA3-GPD-hsvTKCO-ADH-hENT1CO(5x) rad53Δ::KAN sml1Δ::NAT rad5Δ::HPH bar1Δ*	This study
YIN128	*MATa ura3-1::URA3-GPD-hsvTKCO-ADH-hENT1CO(5x) rad53Δ::KAN sml1Δ::NAT exo1Δ::HPH bar1Δ RAD5+*	This study
YIN131	*MATa ura3-1::URA3-GPD-hsvTKCO-ADH-hENT1CO(5x) rad53Δ::KAN sml1Δ::NAT his3::GAL-PRIMPOL bar1Δ RAD5+*	This study
YIN148	*MATa ura3-1::URA3-GPD-hsvTKCO-ADH-hENT1CO(5x) rad53Δ::KAN sml1Δ::NAT his3::GAL-PRIMPOL-AxA bar1Δ RAD5+*	This study
YIN149	*MATa ura3-1::URA3-GPD-hsvTKCO-ADH-hENT1CO(5x) rad53Δ::KAN sml1Δ::NAT his3::GAL-PRIMPOL-CH bar1Δ RAD5+*	This study

*All strains are in the W303 background.

Supplementary Table S2_Primers used in this study

Name	Sequence
RAD5.InFus.fw	CCGGTGTATAAAACAATGAGTCATATTGAACAGGAAGAAAGGAAGAG
RAD5.InFus.rv	TCAATTCAATTCAATCTATTCAAACAGCATCTGGATTTCTTCAATTCT
PRIMPOL.InFus.fw	CCGGTGTATAAAACAATGAATAGAAAATGGGAAGCAAAACTGAAGC
PRIMPOL.InFus.rv	TCAATTCAATTCAATTTACTCTTGTAATACTTCTATAATTAGTTCATCAGGAATTTCATCC
pRS306-G-MUS81.InFus.fw	ATTGAATTGAATTGAAATCGATAGATCAATTTTTTTCTTTTCTCTTTC
pRS306-G-MUS81.InFus.rv	TGTTTTATACACCGGTGTATAGTTTTTTCTCCTTG
HIS3.pRS306.FW.InFus	TAATACAGTTTTTTACTAGTACACTCTATATTTTTTTATGCCTCGGTAATG
HIS3.pRS306.RV.InFus	GAGTGCACCACGCTTCTACATAAGAACACCTTTGGTGGAGGG
pRS306.URA3.FW.InFusion	AAGCGTGGTGCACTCTCAGT
pRS306.URA3.RV.InFusion	TAAAAAACTGTATTATAAGTAAATGCATGTATACTAAACTCACAAATTAG
Mre11-H125N-CRISPR1	GATCATTACCTGATATGCCGAATAGTTTTAGAGCTAG
Mre11-H125N-CRISPR2	CTAGCTCTAAAACTATTCGGCATATCAGGTAAT
Mus81-dd-CRISPR3	GATCGGTTTATGGAGCAAAAAAATGTTTTAGAGCTAG
Mus81-dd-CRISPR4	CTAGCTCTAAAACATTTTTTTGCTCCATAAACC
gRNA.Rad5-I916A.1	GATCGAGTCAAGATGAGAACGATGGTTTTAGAGCTAG
gRNA.Rad5-I916A.2	CTAGCTCTAAAACCATCGTTCTCATCTTGACTC
Rad5Hiran1	GATCCTAAAAGAAGAAGAATATACGTTTTAGAGCTAG
Rad5Hiran2	CTAGCTCTAAAACGTATATTCTTCTTCTTTTAG
